# EGFR oligomerization organizes kinase-active dimers into competent signalling platforms

**DOI:** 10.1038/ncomms13307

**Published:** 2016-10-31

**Authors:** Sarah R. Needham, Selene K. Roberts, Anton Arkhipov, Venkatesh P. Mysore, Christopher J. Tynan, Laura C. Zanetti-Domingues, Eric T. Kim, Valeria Losasso, Dimitrios Korovesis, Michael Hirsch, Daniel J. Rolfe, David T. Clarke, Martyn D. Winn, Alireza Lajevardipour, Andrew H. A. Clayton, Linda J. Pike, Michela Perani, Peter J. Parker, Yibing Shan, David E. Shaw, Marisa L. Martin-Fernandez

**Affiliations:** 1 Central Laser Facility, Research Complex at Harwell, Science and Technology Facilities Council, Rutherford Appleton Laboratory, Harwell Oxford, Didcot, Oxford OX11 0QX, UK; 2 D.E. Shaw Research, New York, New York 10036, USA; 3 Computational Science and Engineering Department, Science and Technology Facilities Council, Daresbury Laboratory, Warrington WA4 4AD, UK; 4 Centre for Micro-Photonics, Faculty of Science, Engineering and Technology, Swinburne University of Technology, Hawthorn, Victoria 3122, Australia; 5 Department of Biochemistry and Molecular Biophysics, Washington University School of Medicine, St Louis, Missouri 63110, USA; 6 Division of Cancer Studies, King's College London, Guy's Medical School Campus, London SE1 1UL, UK; 7 The Francis Crick Institute, Protein Phosphorylation Laboratory, 44 Lincoln's Inn Fields, London WC2A 3LY, UK; 8 Department of Biochemistry and Molecular Biophysics, Columbia University, New York, New York 10032, USA

## Abstract

Epidermal growth factor receptor (EGFR) signalling is activated by ligand-induced receptor dimerization. Notably, ligand binding also induces EGFR oligomerization, but the structures and functions of the oligomers are poorly understood. Here, we use fluorophore localization imaging with photobleaching to probe the structure of EGFR oligomers. We find that at physiological epidermal growth factor (EGF) concentrations, EGFR assembles into oligomers, as indicated by pairwise distances of receptor-bound fluorophore-conjugated EGF ligands. The pairwise ligand distances correspond well with the predictions of our structural model of the oligomers constructed from molecular dynamics simulations. The model suggests that oligomerization is mediated extracellularly by unoccupied ligand-binding sites and that oligomerization organizes kinase-active dimers in ways optimal for auto-phosphorylation *in trans* between neighbouring dimers. We argue that ligand-induced oligomerization is essential to the regulation of EGFR signalling.

## Abstract (web summary)

Epidermal growth factor receptors have been shown to oligomerise upon binding to their cognate ligands. Here, the authors use biochemical, biophysical and cell biology techniques to analyse the structures of these oligomers, and argue that these formations are required for signalling.

Epidermal growth factor receptor (EGFR or HER1/ErbB1) is a cell-surface receptor tyrosine kinase that plays a fundamental role in regulation of cellular metabolism, growth and differentiation^1^. Dysregulation of EGFR or other members of the human EGFR (HER) family (HER2/ErbB2/Neu, HER3/ErbB3 and HER4/ErbB4) is key to the development of various cancers^2^. A number of cancer treatment drugs target HER proteins, and efforts to develop new therapeutic agents targeting these receptors continue^3^.

An EGFR protein consists of a ligand-binding extracellular module and an intracellular module, connected by a single-pass transmembrane helix. The extracellular module consists of four domains and the intracellular module of a short juxtamembrane segment, followed by a tyrosine kinase domain, and a C-terminal tail, where the five key phosphorylation sites (Tyr992, Tyr1045, Tyr1068, Tyr1086 and Tyr1173)^4^ (Fig. 1a) are located. EGFR activation depends on ligand-induced receptor dimerization^5^,^6^ (Fig. 1a), and the structural arrangement of the ligand-induced dimers is well characterized. In such an arrangement, the extracellular domains form a so-called back-to-back dimer^7^,^8^ with the two-ligand-binding sites distal from the dimer interface (Fig. 1a). Dimerization of the extracellular domains, by conformational coupling across the membrane^9^,^10^, promotes formation of catalytically active asymmetric kinase dimers^11^ that auto-phosphorylate the C-terminal tails and initiate downstream signalling.

Pioneering work^5^,^12^ showed that EGFR activation is associated with ligand-induced receptor aggregation, including formation of dimers or oligomers. Although the mainstream of EGFR research has largely focused on a dimerization-dependent activation mechanism, recent analyses suggest that oligomerization also plays a crucial role in EGFR signalling^13^. Ligand-induced EGFR oligomerization was recently characterized^14^ by using single-molecule analysis, showing that mutations blocking oligomerization reduce auto-phosphorylation of EGFR. Despite the potential importance of oligomerization in EGFR signalling, key aspects of EGFR oligomers remain unclear. Here we aim to elucidate the basic architecture, stoichiometry of ligand binding, and functional importance of the ligand-binding induced EGFR oligomers.

## Results

### The geometry of ligand-bound EGFR oligomers

**Figure 1:**
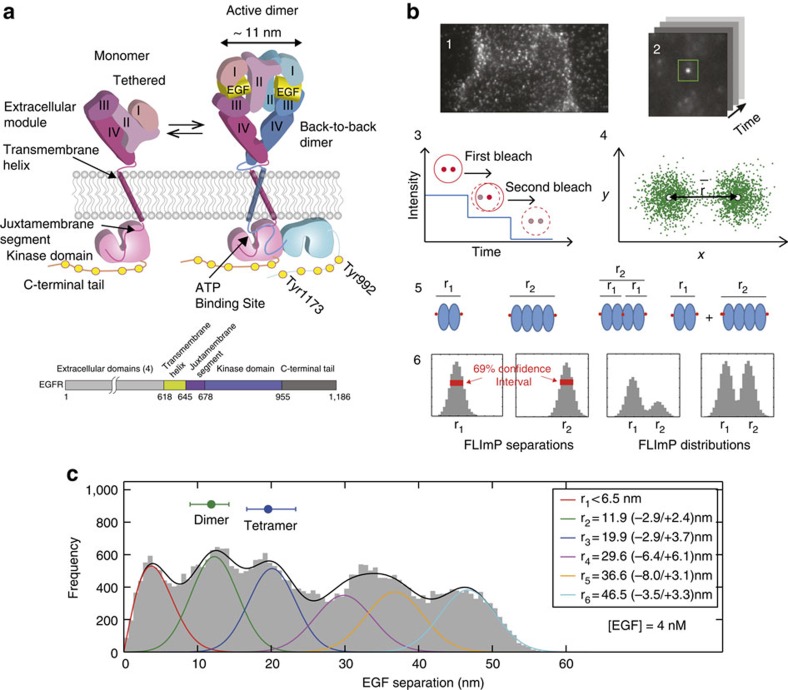
FLImP measurement of pairwise EGF separations. (**a**) Cartoon of an EGFR monomer, a two-ligand active dimer, and an EGFR sequence diagram. (**b**) Steps to determine EGF separations using FLImP^15^: (1) TIRF images are collected from intact cells; (2) spots from individual complexes are tracked to derive intensity time courses; and (3) a spot image of a complex containing two fluorophore-conjugated EGF ligands (red dots) features two intensity levels and decays to zero in two bleaching steps; when one fluorophore bleaches, the centroid position shifts. If more than two steps occur, the lowest two are analysed. (4) A global least-squares seven-parameter-fit is used to identify the best intensity, *x*-*y* positions and the full-width at half-maximum of the point spread function for each fluorophore, from which their separation 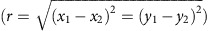 is calculated with a precision determined by the localization error; (5) Example systems of a two-ligand dimer and tetramer, a three-ligand tetramer, and a mixture of a dimer and a tetramer. (6) The empirical posterior distributions (or FLImP measurement) of pairwise ligand separations obtained for each example system with their 69% confidence intervals highlighted. The size of the latter depends on the combined localization errors of the two molecules^15^. FLImP measurements with confidence intervals smaller than the required resolution are retained in a histogram, generating a so-called *FLImP distribution* that is fitted by the sum of a discrete number of Rician peaks (Supplementary Fig. 3a). (**c**) FLImP distribution (grey) of CF640R fluorophore-conjugated EGF on CHO cells (∼10^5^ copies of wild-type EGFR per cell) treated with 4 nM EGF at 4 °C with chemical fixation, compiled from 30 FLImP measurements with confidence intervals <4.8 nm. The distribution is decomposed into a sum of six Rician peaks. Positions and error estimates are shown in the inset. (Details in Supplementary Methods.) The peak positions (and error bars) reflecting the expected dimers and tetramers are marked above the plot. The optimal number of peak components (colour lines) and the best-fit (black line) were determined using a Bayesian information criterion and Bayesian parameter estimation (Supplementary Figs 3b and 4a, and Supplementary Methods).

We used single fluorophore localization imaging with photobleaching (FLImP) to investigate the structure of ligand-induced EGFR oligomers^15^,^16^, measuring discrete pairwise separations between fluorophore-conjugated ligands bound to EGFR complexes (Fig. 1b). Unlike the single-molecule analysis by Kuriyan and colleagues^14^, with fluorophores attached to EGFR intracellularly, we positioned fluorophores extracellularly and FLImP results reflect the extracellular geometry of ligand-bound EGFR complexes. FLImP reports lateral separations between identical fluorophores in the 0–60 nm range (cf. fluorescence resonance energy transfer (FRET), which reports separations between donor/acceptor fluorophores in the range of 2–8 nm). Given the ∼11 nm lateral dimension of active EGFR dimers^7^,^8^, the dimensions of higher-order oligomers are expected to fall in the range appropriate for FLImP experiments. FLImP measurement of the separation of a pair of fluorophore-conjugated ligands bound to an EGFR complex produces an empirical posterior probability distribution of the separation^15^, taking the form of an asymmetric Rice distribution^17^ (Supplementary Fig. 1a–c). The posterior distribution width (or 69% confidence interval) reflects the precision of each pairwise separation measurement, which ultimately depends on signal-to-noise (Fig. 1b). The posteriors with 69% confidence intervals smaller than the required resolution (typically 4–7 nm) are retained and pooled into a histogram (hereafter referred to as FLImP distribution), from which one can derive structural information of EGFR complexes bound to more than one EGF ligand. From a FLImP distribution one can also estimate the proportion of measurements consistent with species of EGFR complexes bound to more than one EGF ligand as an indicator of the relative population of the species.

We first treated Chinese hamster ovary (CHO) cells expressing wild-type EGFR with a physiological concentration (4 nM) of EGF conjugated at its N-terminus with CF640R fluorophore (Biotium) in a 1:1 ratio. At this concentration, ∼10% of available EGF-binding sites are occupied by ligands (Supplementary Fig. 2a). A Bayesian information criterion (see Supplementary Methods) determines the decomposition of the FLImP distribution of EGF separations into six Rician peak components (Fig. 1c). The peak in the range of 0–6.5 nm reflects separations comparable to or below the 4.8 nm resolution of the FLImP measurements; hence the best-fit position of the peak can be susceptible to a well understood bias (Supplementary Fig. 1d,e). For this reason, we quote the confidence interval range but not the best-fit position. The separations in the other five peaks in Fig. 1c fall in the region free from such bias (Supplementary Fig. 1d,e), and their best-fit peak positions (11.9, 19.9, 29.6, 36.6 and 46.5 nm) reflect the underlying pairwise EGF separations[Fig f2][Fig f3].

Based on crystal structures^7^,^8^ and using a dye accessible volume (AV) algorithm to account for the dimensions of the dye and linker^18^ (Supplementary Fig. 2b), we estimate the separation between fluorophores in a two-ligand back-to-back dimer to fall in 12.5±0.3 nm. Thus, the peak at 11.9 (−2.9/+2.4) nm likely represents these dimers (errors are asymmetric because of the inherent asymmetry of the Rice distribution (Supplementary Fig. 1a,c)). Peaks in the 19–50 nm range likely reflect the presence of high order EGFR oligomers at physiological EGF concentrations. Observations suggesting the presence of EGFR oligomers upon ligand binding were previously reported in a diverse group of studies (for example, refs 13, 14, 19, 20). Our finding here is also consistent with single-particle tracking data (Supplementary Videos 1 and 2), which showed clusters of receptors moving together as units in live cells[Fig f4][Fig f5][Fig f6].

In later discussions we show with additional data (Fig. 7a) that the peak in the range of 0–6.5 nm (Fig. 1c) may represent the persistent presence of pre-formed inactive EGFR dimers in spite of exposure to EGF and that these inactive dimers involve the ‘tethered' conformation^21^ of the extracellular domains.

### A structural model of ligand-bound EGFR oligomers

**Figure 2 f2:**
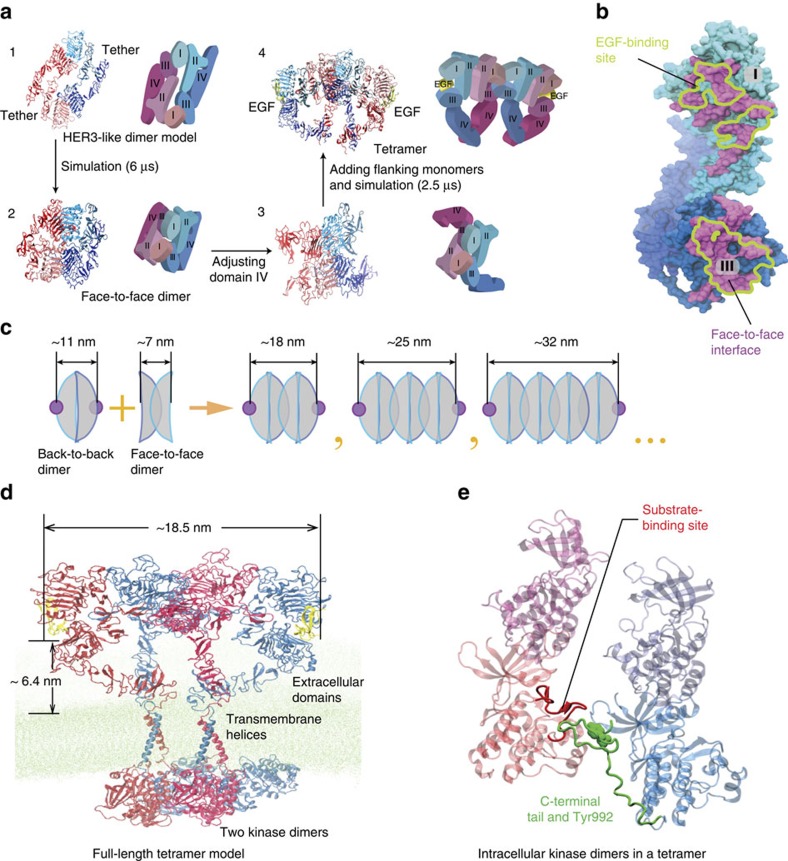
The structural model of EGFR tetramers. (**a**) Key steps in constructing the model of a ligand-bound EGFR tetramer: (1) an initial EGFR dimer model generated using a crystal structure of a HER3 dimer as a template; (2) a face-to-face dimer produced by simulation of the initial dimer model, in which the interaction interface remained unchanged but domains I–III in each monomer departed from the tethered conformation for the conformation seen in the active dimer; (3) domains IV are manually modelled to mimic the conformation of monomers in an active dimer; and (4) a tetramer model constructed by adding two-ligand-bound monomers in back-to-back interactions with the previous dimer. In addition to the ribbons generated using atomic coordinates, cartoon figures are used to illustrate the modeling procedure. (**b**) The site for the face-to-face interaction (purple) and the outline of the largely overlapping EGF binding site at domains I and III. (**c**) A diagram illustrating the open-ended oligomerization scheme for EGFR extracellular domains based on repeating the back-to-back and the face-to-face interactions. (**d**) The full-length structural model of an EGFR tetramer as a dimer of active dimers assembled by the face-to-face interactions. The predicted separation between the N-termini of the two EGF ligands and the average EGF-membrane distance are marked. The coordinates of the model are available in Supplementary Data. (**e**) The arrangement of the two intracellular active kinase dimers in the tetramer model, by which the phosphorylation site Tyr992 (green) of one receptor is positioned in the proximity of the active site (red) of a kinase domain from the neighbouring dimer.

In parallel to the FLImP experiments, we attempted to construct a structural model of EGFR oligomers using long-timescale molecular dynamics (MD) simulations. Conjecturing that lattice contacts of existing crystal structures of EGFR and its homologues might reveal previously unidentified oligomerization interfaces, we examined crystal contacts in all published structures of the extracellular domains of HER family members. We found a crystallographic dimer of HER3 (PDB 1M6B (ref. 22)), structurally unrelated to the back-to-back dimer and featuring an extensive dimer interface (18.8 nm^2^ in area, as compared with 14.7 nm^2^ for the back-to-back dimer (PDB 1MOX)^8^). Unlike the back-to-back dimer interface, which is located on domain II, the crystallographic dimer interface is located on domains I and III. Since back-to-back and crystallographic dimer interfaces do not overlap, we hypothesized that EGFR can oligomerize by making use of both interfaces simultaneously. As a first step in constructing an EGFR oligomer model along these lines, we built an EGFR dimer model using the HER3 crystallographic dimer as a template (Fig. 2a). In this dimer, two monomers assume the tethered conformation and the dimerization interface is predominantly between domain I of one monomer and domain III of the other.

Although dimer interfaces between domains I on one side and domains III on the other were well maintained in an MD simulation (6 μs), both monomers departed from the tethered conformation in a concerted fashion (Supplementary Fig. 5a,b). This simulation produced a face-to-face extracellular dimer model, in which each monomer's conformation is similar to that in the back-to-back dimer, but the dimer interface is entirely different. Two back-to-back dimers can be assembled into a tetramer using the face-to-face interface at domains I and III, which is located at the opposite side of the back-to-back interface at domain II (Fig. 2a). Importantly, because the face-to-face dimer interface largely overlaps with the ligand-binding interface (Fig. 2b), such a model implies that ligand binding and the face-to-face dimer interaction are in competition with one another.

This tetramer model can in principle be extended into higher-order oligomers by incorporating additional dimers and repeating the face-to-face interaction. An oligomer of such structures can bind two EGF ligands at most, as all EGF-binding sites are engaged in the face-to-face interaction except for the monomers at the two open ends (Fig. 2c). Separations between receptor-bound EGF ligands predicted by this model are 18.5 nm for a tetramer (Fig. 2d), 26 nm for a hexamer, 33.5 nm for an octamer and 41 nm for a decamer. The oligomer model thus predicts that peaks in a FLImP distribution of pairwise EGF separations for a mixture of EGFR oligomers should be separated by ∼7.5 nm. Given that the fluorophore diameters add 1.1±0.5 nm to the 18.5 nm separation between the two ligands bound to a tetramer (Supplementary Fig. 2c), the separation at 19.9 (−2.9/+3.7) nm suggested by the FLImP distribution at 4 nM EGF (Fig. 1c) is well within that anticipated by the tetramer model. Decomposition of the FLImP distribution also exhibits peaks at 29.6, 36.6 and 46.5 nm, remarkably consistent with the predictions of the hexamer, the octamer and the decamer models, especially considering the 4.8 nm resolution of the FLImP experiment.

**Figure 3 f3:**
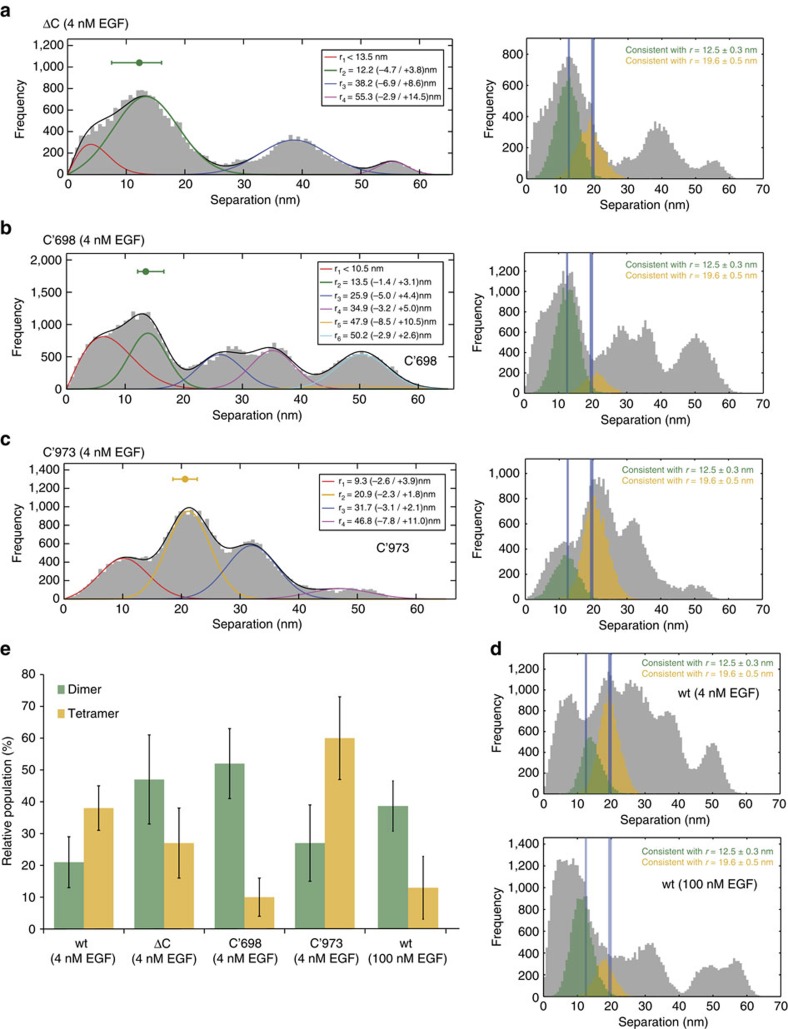
Pairwise EGF separations in three EGFR deletion mutants. (**a**) (left) FLImP distribution (grey) of pairwise EGF separations on CHO cells expressing ΔC-EGFR treated with 4 nM EGF. The distribution is fitted (black line) with a sum of four Rician peaks (colour lines). The number of peaks used was determined using a Bayesian information criterion. The best-fit positions of the peaks and error bars are shown in the inset. The errors in the fit were calculated as described in Supplementary Methods. (right) The FLImP distribution (grey) and the distributions (green or yellow) compiled from the FLImP measurements whose ranges of 69% confidence overlap with the ranges of EGF separations of the expected dimer (12.5±0.3 nm) or tetramer (19.6±0.5 nm), which are indicated by the vertical blue lines. (**b**) Similar to **a** for C'698 EGFR. (**c**) Similar to **a** for C'973 EGFR. Cells stably expressed each mutant at an expression level of ∼10^5^ copies per cell. (**d**) Similar to **a** for the wild-type receptor respectively treated with 4 and 100 nM EGF. The numbers of FLImP measurements included in each distribution are 40 (ΔC-EGFR), 44 (c'698-EGFR), 33 (c'973-EGFR), 51 (wild type receptor at 4 nM EGF), and 37 (wild type receptor at 100 nM EGF); the confidence interval for each included FLImP measurement is 6 nm (**d**) or 7 nm (**a**–**c**). (**e**) Relative populations of the dimers and the tetramers, determined from the FLImP measurements shown in **a**–**d**, right hand panels. For each construct, the dimer percentage is estimated by the ratio of the green integral area to the integral area of all FLImP measurements whose 69% confidence overlaps with the dimer-tetramer region (0–20.1 nm). Tetramer populations are calculated in the same way, using the yellow instead of the green integral area. Error bars were calculated by bootstrap-resampling the data 1,000 times with replacement and repeating the analysis.

We extended the extracellular tetramer model to obtain a full-length model of the EGFR tetramer (Fig. 2d and Supplementary Data), based on the previously reported full-length dimer model^9^,^10^ and the naive assumption that the tetramer is essentially a dimer of the active dimers. When this model was tested by MD simulations of up to 40 μs, the tetramer model remained stable, and the two intracellular kinase dimers formed direct interactions with one another (Fig. 2e). (For model coordinates see Supplementary Data). Despite this model, how the intracellular modules interact within an EGFR oligomer remains obscure. Our mutagenesis studies suggest that intracellular interactions are crucial to EGFR oligomerization. The FLImP distribution of the wild-type receptor at 4 nM EGF (Fig. 1c) exhibits peaks, respectively, corresponding to underlying dimers (11.9 nm) and tetramers (19.9 nm). However, at the same EGF concentration, the tetramer peak is not resolved in the FLImP distribution of ΔC-EGFR (the entire intracellular module is truncated) or C'698-EGFR (the juxtamembrane segment remains) (Fig. 3a,b), suggesting a decrease of the tetramer population relative to the dimers. This is quantitatively shown by the reduction in the proportion of FLImP measurements consistent with the expected 19.6±0.5 nm tetramer position (Fig. 3e). On the other hand, the FLImP distribution of c'973-EGFR, where only the C-terminal tail is truncated, shows a relative increase of the tetramer population (Fig. 3c,e).

### Oligomerization promotes EGFR auto-phosphorylation

While it is well established that the active kinase dimer switches on kinase activity by stabilizing one of the kinase domains in an enzymatically active conformation^11^, the mechanism that ensures substrate access to the activated kinase domain in EGFR auto-phosphorylation is unclear. In the simulation of the tetramer model, the two active kinase dimers arrived at an intriguing arrangement in which a C-terminal phosphorylation site (Tyr992) from one dimer was positioned adjacent to the substrate-binding site of the activated kinase of the other (Fig. 2e and Supplementary Fig. 5d), hinting at the possibility that EGFR auto-phosphorylation may be realized in the context of tetramers or higher-order oligomers, occurring *in trans* between two neighbouring kinase dimers. A large part of the C-terminal tail was found to be auto-inhibitory to EGFR phosphorylation^23^ and deletion of the C-terminal tail (C'973 mutant; Fig. 3c) promotes tetramer formation. These two findings together also suggest a crucial role of oligomerization in EGFR auto-phosphorylation.

**Figure 4 f4:**
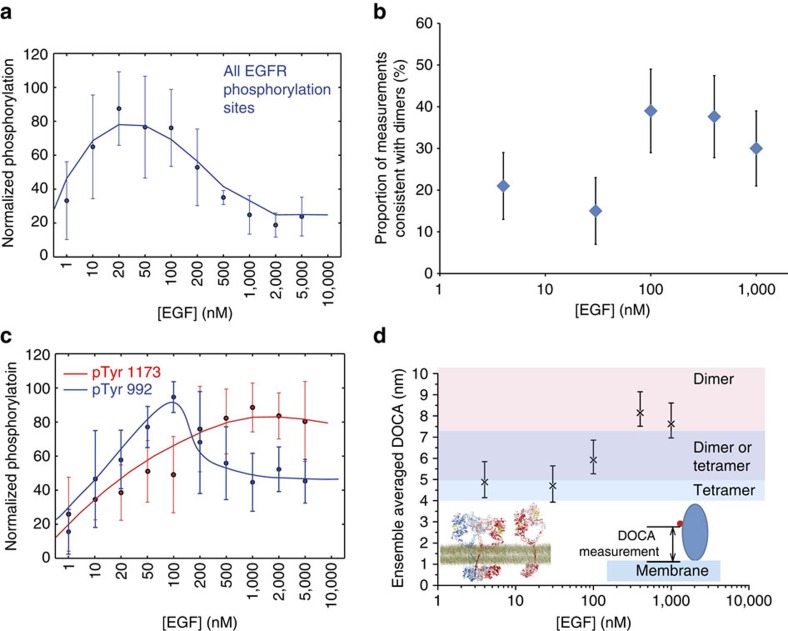
Dependence of EGFR phosphorylation and oligomerization-related structural parameters on ligand concentration. (**a**) Western blot measurement of wild type total EGFR auto-phosphorylation in CHO cells exposed to increasing concentrations of EGF. The monoclonal pan-phosphotyrosine antibody 4G10 was used in the measurements. Data points and standard deviations are derived from the average of three independent measurements (examples western blot images shown in Supplementary Fig. 6c). (**b**) Similar to Fig. 3e, estimate of the relative population of EGFR dimers. The estimates are based on the wild-type FLImP distributions at varying EGF concentrations (Fig. 3e and Supplementary Fig. 7). Errors are calculated as in Fig. 3. (**c**) Western blot measurements of phosphorylation of Tyr1173 and Tyr992 in CHO cells exposed to increasing EGF concentrations. Data points and error bars (s.d.) are derived from the average of four independent measurements (examples shown in Supplementary Fig. 6a,b). For the Tyr992 data, *P* values were calculated using Student's *t*-test to determine whether measured phosphorylation at high EGF concentrations was significantly different from the maximum phosphorylation value measured at 100 nM EGF. *P* values are: 50 nM EGF, *P*=0.058; 200 nM EGF, *P*=0.173; 500 nM EGF, *P*=0.027; 1,000 nM EGF, *P*=0.014; 2,000 nM EGF, *P*=0.011; 5,000 nM EGF, *P*=0.009. (**d**) The DOCA between EGFR-bound EGF molecules and the membrane, derived from FRET measurements shown in Supplementary Fig. 8. DOCAs were obtained from 1,000 bootstrap data sets (that is, data sets resampled with replacement). The error bars are the standard deviations of the bootstrap means. Simulations of the tetramer (Fig. 2d and Supplementary Fig. 5c) and dimer^9^ (bottom left inset) predict a DOCA of ∼5 nm for oligomers and ∼7.5 nm for dimers.

According to our structural model, an oligomer of 2N receptors can bind to a maximum of two EGF ligands (N:1 stoichiometry) (Fig. 2c), and an oligomer is a collection of back-to-back dimers assembled by face-to-face interactions at unoccupied ligand-binding sites. It follows that at sufficiently high EGF concentration, ligand–receptor interactions may out-compete the face-to-face interactions and break oligomers into dimers. If indeed EGFR auto-phosphorylation occurs in the context of oligomers, one should expect high EGF concentrations to reduce EGFR auto-phosphorylation. To test this somewhat counterintuitive prediction, we treated CHO cells expressing wild-type EGFR with increasing concentrations of EGF and measured the level of EGFR auto-phosphorylation using an antibody (4G10). In these cells, which do not express any phosphorylatable proteins of similar size to EGFR^24^, 4G10 specifically recognizes all EGFR phosphotyrosines. The data in Fig. 4a show a biphasic EGF dependence of EGFR auto-phosphorylation, peaking at ∼30 nM EGF and, as anticipated, returning to the basal level at 1–5 μM EGF.

To investigate whether high EGF concentrations break oligomers into ligand-bound dimers, we performed FLImP experiments at increasing concentrations of EGF on CHO cells expressing wild-type EGFR (Supplementary Fig. 7). If EGF out-competes face-to-face interactions, at higher EGF concentration one would expect an increase in pairwise EGF separations consistent with back-to-back dimers. This is indeed what we found (Fig. 4b). At EGF concentrations beyond 100 nM, at which EGFR auto-phosphorylation begins to return to the basal level, there is an overall increase in separations consistent with dimers (12.5±0.3 nm). This finding supports the notion that the decline in EGFR auto-phosphorylation is associated with disruption of oligomerization.

To further characterize EGFR auto-phosphorylation, we examined phosphorylation of Tyr1173 and Tyr992. Tyr1173 is the first of the five C-terminal tyrosine phosphorylation sites to be phosphorylated after EGF binding^25^. Of these sites, Tyr1173 is also closest to the C-terminus (most distal to the kinase domain in sequence) and is preceded by an unstructured loop of over 200 residues (Fig. 1a). We reasoned that the length and flexibility of the loop may allow Tyr1173 access to an activated kinase domain in *cis* within an EGFR dimer and if so, Tyr1173 phosphorylation may be unique in its independence from EGFR oligomerization. Consistent with this hypothesis, using a pTyr1173-specific antibody we showed that, in contrast to the overall EGFR auto-phosphorylation (Fig. 4a), Tyr1173 phosphorylation plateaus at ∼500 nM EGF and remains high up to 5 μM EGF (Fig. 4c).

Tyr992, on the other hand, is the tyrosine phosphorylation site most proximal to the kinase domain in sequence, and topologically unique among the other EGFR C-terminal tyrosines^26^. We reasoned that the phosphorylation of Tyr992, unlike Try1173, may have to be realized in *trans* between neighbouring dimers within an oligomer (Fig. 2e). Indeed the phosphorylation of Tyr992 shows a decrease at EGF concentrations beyond 100–200 nM (Fig. 4c), where the fraction of dimers is higher (Fig. 4b). This decrease is statistically significant, as shown by *P* values given in the fig. 4 caption. Since pTyr992 and pTyr1173 recruit different effectors^27^ and elicit different downstream signals, these signals may respond differently to a shift in EGF concentration.

Another simulation prediction is that, on average, an oligomer-bound ligand is positioned closer to the membrane than a dimer-bound ligand. In our molecular model of the oligomer, the extracellular modules of two EGFR dimers engaging in face-to-face interactions with one another must tilt in opposite directions. In comparison, the extracellular module of an active EGFR dimer fluctuates around an upright orientation. In our previous simulation (4.4 μs) of the back-to-back dimer^9^, EGF ligands are at an average distance of 7.7±2.7 nm from the membrane; in another simulation carried out in this study (6.4 μs), the distance was 7.5±2.5 nm. By contrast, the average distance of the N-termini of tetramer-bound ligands to the membrane was smaller, 6.4±0.9 nm in one simulation of our tetramer model and 5.0±0.95 nm in another (Supplementary Fig. 5c).

We employed FRET to measure the distance of the closest approach (DOCA)^28^ between donor Alexa 488-conjugated EGF ligands and acceptor DiD probes embedded in the membrane (Supplementary Fig. 8a–e). If high EGF concentrations indeed result in breaking EGFR oligomers into dimers, we expect EGF-membrane distance to increase at high EGF concentrations. As shown by DOCA, the average EGF-membrane distance rises from 4.8±0.9 nm at 4 nM EGF to 8±0.8 nm at 400 nM EGF and 7.5±0.8 at 1 μM EGF (Fig. 4d). Importantly, the increase of EGF distance to the membrane is in line with the relative increase in FLImP readings of EGF separations consistent with dimers (Fig. 4b) at the expense of readings consistent with tetramers.

**Figure 5 f5:**
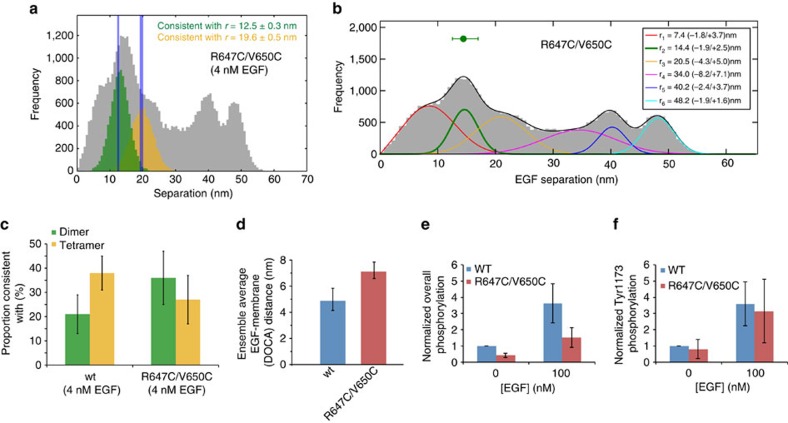
Pairwise EGF separations and phosphorylation of R647C/V650C. (**a**) FLImP distribution (grey) of pairwise EGF separations on CHO cells expressing the R647C/V650C-EGFR mutant at a level of ∼10^5^ copies per cell. The separations whose confidence intervals overlap with the 12.5±0.3 nm (green) or 19.6±0.5 nm (yellow) expected dimer and tetramer interval are shown. The expected intervals are indicated by the vertical blue lines. The distribution includes data from 40 FLImP measurements with confidence intervals <6 nm. (**b**) Peak decomposition (colour lines) and best-fit (continuous black line) of the FLImP distribution. The optimal number of underlying peak components (colour lines) and the best-fit (black line) were determined using a Bayesian information criterion. The best-fit positions of the peaks and error bars are shown in the inset. (**c**) Similar to Fig. 3e, an estimate of the relative populations of dimers and tetramers. (**d**) Comparison of EGFR R647C/V650C and the wild-type receptor in terms of DOCA distances between receptor-bound EGF molecules and the membrane at 4 nM EGF derived from FRET measurements shown in Supplementary Fig. 8f. (**e**) Overall and (**f**) Tyr1173-specific phosphorylation of EGFR R647C/V650C compared with the wild type at 100 nM EGF. The data points and error bars (s.d.) are obtained from three independent measurements. An example is shown Supplementary Fig. 6d.

**Figure 6 f6:**
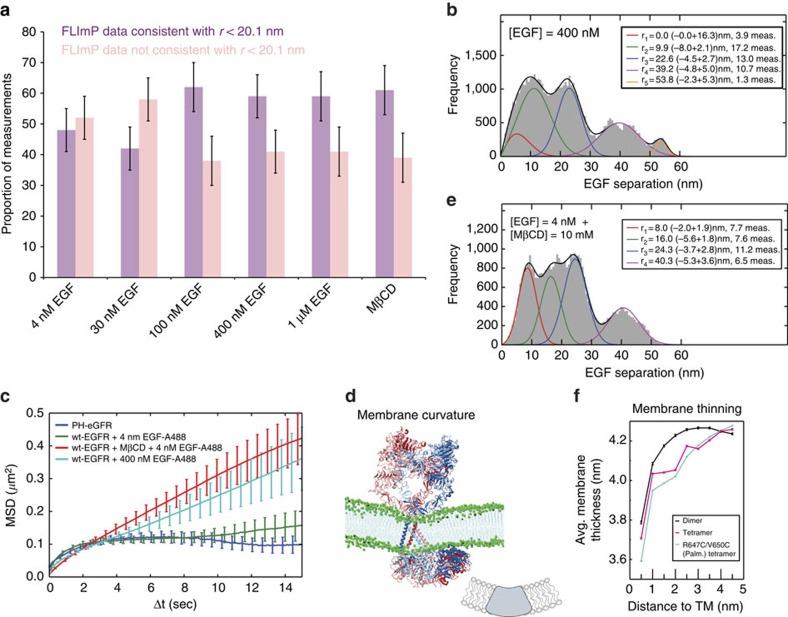
Membrane bending and EGFR oligomerization. (**a**) Percentage of separations whose 69% confidence intervals overlap with the dimer/tetramer range (*r*<20.1 nm) or do not (*r*> 20.1 nm), estimated by the ratio of the integral area of interest (purple or salmon in FLImP distributions, Supplementary Fig. 7) to the integral area of the distribution. (**b**) FLImP distribution (grey) of pairwise separations of fluorophore-conjugated EGF on CHO cells expressing ∼10^5^ copies of wild-type EGFR treated with 400 nM EGF (46 measurements). The distribution is fitted (black line) with a sum of five Rician peaks (colour lines). Best-fit positions and error bars shown in the inset. (**c**) (green) MSD plot from single-particle tracks of wild-type EGFR complexes on live CHO cells at 37 °C labelled with Alexa 488-conjugated EGF; (dark blue) MSD plot from single-particle tracks of PIP_2_ labelled with a PLCδ1-Pleckstrin homology (PH) domain (PH-eGFP) fusion construct which specifically binds PiP_2_ (ref. 75), transfected on CHO cells expressing wild-type EGFR. MSD plots include data from ∼>10^3^ tracks and three biological repeats. Bootstrap-estimated errors (vertical line) are shown. Linear MSD plots suggest Brownian motion; concave-down MSD plots suggest confinement at the plasma membrane^76^. (**d**) The negative curvature of the membrane local to an EGFR tetramer in simulation. The cartoon illustrates the membrane-bending effect of the N-terminal dimers of EGFR transmembrane helices as a hydrophobic wedge. (**e**) FLImP distribution (grey) of pairwise separations of fluorophore-conjugated EGF on the surface of CHO cells expressing ∼10^5^ copies of wild-type EGFR pre-treated with 10 mM MβCD, exposed to 4 nM EGF (33 measurements). Best-fit positions and error bars are shown in the inset. (**f**) Membrane thickness (*Y*-axis) as a function of distance to the transmembrane helices (*X*-axis) in simulations of the wild-type active dimer and tetramer, and the palmitoylated R647C/V650C tetramer. The membrane thickness is indicated by the average separation between the two sheets of phosphorus atoms of the two lipid layers. The wild-type tetramer (red line) exhibited a more pronounced membrane-thinning effect. The data are plotted as averages and standard error of the mean over frames of the simulations.

The R647C/V650C mutant, which is palmitoylated in the juxtamembrane segment at the two cysteines, is another example where impaired auto-phosphorylation^29^ coincides with an increase in the FLImP readings of EGF separations consistent with dimers (Fig. 5a–c), and in the average ligand–membrane distance (7.1±0.6 nm) (Fig. 5d). The FLImP distribution of R647C/V650C at 4 nM EGF, reminiscent of the wild-type distribution at 1 μM EGF (Supplementary Fig. 7d), suggests that oligomerization is hindered by the double mutation. We further showed that, similar to the effect of high EGF concentration on the wild-type, R647C/V650C palmitoylation impairs overall EGFR auto-phosphorylation but not phosphorylation of Tyr1173 (Fig. 5f and Supplementary Fig. 6d for western blot images). Simulation studies suggest that the effect of R647C/V650C may arise from the extension of the hydrophobic length of the transmembrane helices due to palmitoylation (Fig. 6f).

Our oligomer model correctly anticipates that high EGF concentrations would lead to the breaking of EGFR oligomers into two-ligand dimers (Fig. 3d and Supplementary Fig. 7b). The model, however, does not explain the re-emergence of the peaks of large EGF separations at higher than physiological EGF concentrations (400 nM and 1 μM; Supplementary Fig. 7c,d). Formation of oligomers at non-physiological concentrations has previously been reported^14^. We cannot provide a definitive explanation for this phenomenon, but speculate that other forms of EGFR oligomers with different EGF stoichiometry may dominate at EGF concentrations far beyond physiological. This is supported by two findings. First, the oligomers formed at EGF concentrations >100 nM are not only smaller than those formed at <30 nM EGF (Fig. 6a), but also display a different geometry (Fig. 6b and Supplementary Fig. 7b–d). Secondly, unlike EGFR oligomers at low EGF concentrations, those at high concentrations are not confined within plasma membrane compartments. EGFR is known to interact with phosphatidylinositol 4,5-bisphosphate lipids (PIP_2_) (ref. 20). The similarity of mean square displacement (MSD) plots from tracking EGFR and tracking PIP_2_ on the cell surface (Fig. 6c) suggests that EGFR oligomers formed at 4 nM EGF are confined in (PIP_2_)-enriched plasma membrane regions. In contrast, EGFR oligomers formed at 400 nM EGF are not confined to PIP_2_-enriched regions. These results suggest that EGFR oligomers at low- and high EGF concentrations occupy different plasma membrane regions, raising the possibility that plasma membrane environment may influence EGFR oligomer geometry.

### Membrane bending and EGFR oligomerization

Our simulations of the tetramer model showed an intriguing local thinning of the membrane and spontaneous formation of negative membrane curvature centred on the transmembrane helices (Fig. 6d,f) organized into two N-terminal helix dimers (dimer interfaces near the N-terminus of the helices). The transmembrane dimers organized by oligomers may insert in the membrane as hydrophobic wedges (Fig. 6d) are known to promote local bending of the membrane^30^. Extracellular and intracellular modules of the oligomers also form intimate contacts with the membrane surfaces, potentially exerting a sculpting effect on the membrane. It is conceivable that clustering of EGFR oligomers may induce membrane bending, and conversely, interference with membrane bending may affect EGFR oligomerization.

Previous work showed a complex interplay between membrane curvature and cholesterol distribution in lipid bilayers^31^. Of particular interest, the presence of cholesterol is found to help induce negative membrane curvature^32^. To test coupling between membrane curvature and EGFR oligomerization, we used FLImP to measure EGF pairwise separations at 4 nM EGF in the presence of 10 mM methyl-β-cyclodextrin (MβCD), which removes cholesterol from the membrane^19^,^33^. Addition of MβCD significantly altered the FLImP distribution, suggesting a changed oligomerization pattern. Intriguingly, the FLImP distribution at 4 nM EGF with MβCD (Fig. 6e) is remarkably similar to the distribution at 400 nM EGF without MβCD (Fig. 6b). A quantitative analysis shows that oligomers formed at 4 nM EGF with MβCD are smaller than those at 4 and 30 nM EGF in the absence of MβCD (Fig. 6a). Consistent with this, the MSD plot derived from tracking EGFR oligomers formed at 4 nM EGF in cells pre-treated with MβCD shows a reduction in confinement of receptor complexes in PIP_2_-enriched regions of the membrane of comparable magnitude to that found for 400 nM EGFR in the absence of MβCD (Fig. 6c). This again suggests that plasma membrane environments may influence the geometry of EGFR complexes.

The MβCD results are consistent with cooperativity between membrane bending and EGFR oligomerization, which may help clustering of active EGFR oligomers in clathrin-coated pits^34^ and internalization of EGFR, a key mechanism for negative feedback in EGFR signalling^35^. Such a scenario would provide an explanation for the connection of EGFR internalization and enhanced EGFR oligomerization. Further research is required to fully establish this connection and to clarify the molecular mechanism behind it.

### Inactive EGFR dimers of the tethered conformation

**Figure 7:**
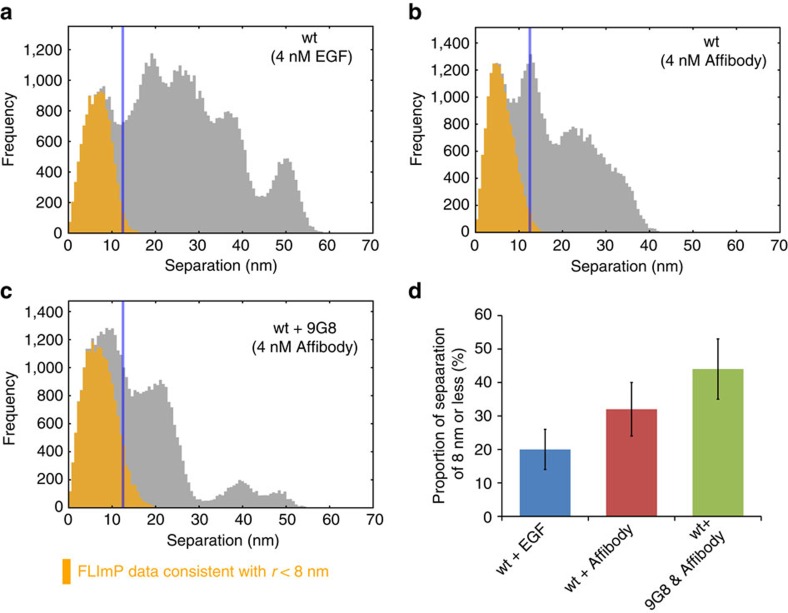
The tethered ectodomain signature in FLImP distributions. (**a**) FLImP distribution (grey) of pairwise separations of fluorophore-conjugated EGF bound to EGFR on CHO cells treated with 4 nM EGF (identical data as shown in Fig. 3d) and the distribution (yellow) compiled from all FLImP measurements whose 69% confidence interval overlaps with the range of 0–8 nm for the inactive dimers. (**b**) Similar to **a**, FLImP distribution of cells treated with 4 nM anti-EGFR Affibody. (**c**) Similar to **b** on cells pre-treated with 200 nM 9G8 nanobody and 4 nM Affibody. The distributions in **b**,**c** contain data from 37 and 33 FLImP measurements, respectively. The expected range of separations for dimers is indicated by the vertical blue lines. (**d**) Estimate of the relative population of the inactive dimers as indicated by ratio of the yellow integral area to the corresponding total grey integral area. Errors are calculated as described in Fig. 3.

As noted earlier, the FLImP distribution at 4 nM EGF exhibits a short peak in the range of separations of <6.5 nm (Figs 1c and 7a) that cannot be explained by our model (Fig. 2d). Similar short EGF separations were previously observed using FRET (for example, refs 13, 36). Echoing this observation, the FLImP distribution of CF640R fluorophore-conjugated anti-EGFR Affibody antagonist^37^ showed a peak in the range of separations of <8 nm (Fig. 7b). As the Affibody inhibits EGFR activity and competes with EGF for the same binding site^37^,^38^, this Affibody peak likely reflects a set of complex structures of inactive receptors. Quantum-dot-based optical tracking showed that in the absence of EGF, EGFRs form inactive or ‘pre-formed' dimers of finite lifetime that are primed for ligand binding^39^. Modelling based on MD proposed an active-like extracellular structure of the inactive dimer^9^, while other analysis^40^ argues that the extracellular domains of the inactive dimers are structurally diverse and the constituent monomers may assume the tethered conformation. The corresponding peak in the FLImP distribution of EGF ligands suggests that such inactive EGFR complexes persist despite the presence of EGF. When 200 nM 9G8 anti-EGFR nanobody is added to the Affibody treatment, the population of short Affibody separations detected by FLImP increases (Fig. 7c,d). Because 9G8 binding to EGFR is non-competitive with Affibody binding (Supplementary Fig. 9) and is selective to the tethered conformation (Fig. 1a)^41^, the short Affibody separations likely reflect inactive EGFR dimers with tethered conformation. The Affibody FLImP distributions exhibit a second prominent peak at 12.0±1.6 nm (Supplementary Fig. 4b and Fig. 7b), indicative of another form of inactive dimers. The diminishment of this peak by 9G8 (Fig. 7c) suggests that the alternative inactive dimer is not tethered.

### Control experiments against FLImP artefacts

Receptors must be completely immobilized on cells at the nanometre scale to achieve resolutions below 10 nm. Our FLImP experiments therefore use chemical fixation (3% paraformaldehyde + 0.5% glutaraldehyde), which is widely used and compatible with the total internal reflection fluorescence (TIRF) illumination method used for FLImP (Fig. 1b). Chemical fixation could in principle introduce crosslinking artefacts. To investigate this possibility, in a previous report^16^ we showed that FLImP reproduced the breaking of higher-order EGFR oligomers into dimers upon receptor downregulation by phorbol myristate acetate (PMA) activation of protein kinase C (PKC), a signalling effector downstream to EGFR. Moreover, we showed that PKC inhibitor bisindolylmaleimide-I (BM-I) cancels out the effect of PMA treatment^16^. As PMA does not abolish the ability of EGFR to interact at the plasma membrane with one another (Supplementary Fig. 10a–e), the results of our previous PMA/BM-I control experiments are inconsistent with an overriding chemical crosslinking artefact in our FLImP measurements.

In a separate report^42^, we showed that FLImP distributions change as expected upon cholesterol removal from the membrane. Here in this study we showed clustering of receptors moving together in units larger than dimers on live cells (Supplementary Videos 1 and 2). Moreover, we showed that high EGF concentrations (Figs 4 and 6) and EGFR mutations (Figs 3 and 5) can disrupt oligomerization. The number and brightness of receptor particles detected on the cell surface remain constant before and after treatment with crosslinking reagent (Supplementary Fig. 11a) and, as shown by photobleaching image correlation spectroscopy^43^, sizes of EGFR clusters remain unchanged after fixation (Supplementary Fig. 11b). These findings, combined with the effects of PKC inhibitors on EGFR oligomerization, strongly argue against the possibility of significant crosslinking artefacts in the FLImP experiments.

We employed low-temperature incubation (∼4 °C) to block EGFR-mediated coated pits from entry into the cell^44^, a process that begins seconds after the ligand stimulus^45^ at physiological temperature. Published data suggest that EGF binding to EGFR proceeds as normal at 0 °C (ref. 46), and that low temperature does not change EGFR localization^33^ nor significantly affect EGFR phosphorylation and signalling^20^,^45^. Exposing cells to low temperatures can in principle depolymerize the cytoskeleton^47^ and alter the size of plasma membrane domains^48^, both of which may disrupt EGFR interactions with one another and introduce artefacts. We found that depolymerizing the F-actin and microtubule networks by treating cells with Latrunculin A^49^ or Nocodazol^50^, or altering the size of plasma membrane domains by treating cells with Nystatin or MβCD (ref. 33), have significant effects on the size of the plasma membrane compartments in which EGFR diffuses (Supplementary Fig. 10a), the rate of EGF-induced oligomerisation (Supplementary Fig. 10b), and the half-life of EGFR complexes (Supplementary Fig. 10c). In contrast, the size of the membrane compartments, the rate of EGF-induced oligomerization, or the half-life of EGFR complexes after low-temperature incubation are statistically indistinguishable from those after incubation at 37 °C (Supplementary Fig. 10a–e), suggesting that low-temperature incubation used in our experiments does not introduce significant artefacts. The only noticeable effect of low temperature is a reduction in the diffusion rate of EGFR (Supplementary Fig. 10d), as reported previously^44^.

We then used Affibody FLImP experiments as another layer of control against potential artefacts of low-temperature incubation. Given that Affibody does not activate the receptor^42^ (Supplementary Fig. 9), and therefore does not induce receptor mediated endocytosis, we compared the FLImP distribution obtained after low-temperature incubation to that with Affibody probe at room temperature (∼21 °C), at which actin polymerization kinetics *in vitro* proceed as normal^47^. Reassuringly, the FLImP distributions were quantitatively indistinguishable (Supplementary Fig. 12), again suggesting that the low temperature does not introduce significant artefacts.

We next enquired whether low-temperature incubation was sufficient to alter the cytoskeleton. To test this, we used fluorescence microscopy under epi-illumination and TIRF illumination to compare the inner and cortical actin cytoskeleton in cells incubated at 4^o^C for 1 h with those of cells not subjected to this treatment. As shown in Supplementary Fig. 13, the distribution and abundance of actin filaments is indistinguishable.

While in principle it remains possible that certain artefacts may have escaped our scrutiny and affected our results, we believe that the substantial body of controls (Supplementary Figs 10–13) in this work and previous findings by us^42^,^51^ and others (for example, refs 20, 33, 45, 46) rule out any significant artefacts associated with chemical crosslinking or low incubation temperature adopted in the FLImP protocol.

## Discussion

In summary, a body of data acquired from a multi-technique study (involving high-resolution FLImP, FRET, long-timescale MD simulations, single-particle tracking and biochemical assays) suggests that efficient EGFR auto-phosphorylation requires the formation of at least EGFR tetramers. While ligand-induced dimerization is fundamental in ensuring the activation of EGFR kinases, oligomerization is likely also essential in ensuring efficient substrate access to the activated kinases. We propose a structural model of EGFR oligomers by which an oligomer binds two EGF ligands, involving a previously not discussed interaction of two neighbouring EGFR molecules mimicking their interaction with EGF. Results on intracellular mutants of EGFR suggest that interactions of the intracellular kinase domains are crucial to oligomerization, but further high-resolution investigations are needed for a clear understanding of these interactions.

It is tempting to speculate that the structural basis of EGFR oligomerization we propose may also be applicable to other HER family members. Although the formation of HER2/HER3 dimers appears to be sufficient for HER3 phosphorylation, it was suggested that phosphorylation of HER2 may require higher-order HER2/HER3 complexes^52^. Recently, it was also suggested that higher-order oligomerization may explain why phosphorylation of the C-terminal tail of HER receptors is always asymmetric, independent of the sequence of the tail^23^. Indeed, the tail of the activator is always more phosphorylated, and this may not be accounted for by contacts within an asymmetric dimer, while higher-order oligomerization can sterically determine the access of the tail to activated kinases in other dimers^23^. It was further suggested that HER3 oligomerization involves two distinct interfaces: one where interactions are independent of ligand binding and another where interactions are disrupted by it^53^. This is intriguingly reminiscent of the back-to-back and face-to-face interactions involved in our model of EGFR oligomers. Moreover, our results imply that ligand binding at higher concentrations is energetically less favourable because it disrupts face-to-face interactions in oligomers. This may be an important factor contributing to the negative cooperativity of EGFR ligand binding^54^.

This study suggests biphasic EGFR signalling based on an intricate balance of EGFR dimers and oligomers modulated by ligand concentration and binding affinity. The model proposes that a relatively low ligand concentration is sufficient to elicit a high degree of EGFR auto-phosphorylation, explaining the ‘super-stoichiometric' signalling behaviour displayed by EGFR at low EGF concentrations. (The number of phosphorylated EGFR molecules is approximately threefold larger than that afforded by the number of bound EGF molecules^55^ and the Grb2:EGFR recruitment stoichiometry can be as high as 3.5:1 (ref. 56).) In contrast, high EGF concentration hinders EGFR oligomerization and suppresses Tyr992 phosphorylation (but not Tyr1173). Instead of a simple positive correlation between signalling and ligand concentration, there could be at least two distinct responses to stimulus and differential biological consequences: one associated with Tyr1173 phosphorylation, and another associated with Tyr992 phosphorylation, as suggested by proteomic studies^27^ and by results that associate oligomerization closely with Tyr992 phosphorylation and PI3K activation but not with distal C-terminal phosphorylation of ERK^14^. Indeed, it was also reported that, while low EGF concentrations can activate most canonical pathways (for example, Erk, Akt, Shc1, CrkL, Cbl and Gab1)^57^, high EGF concentrations trigger pathways of ubiquitination and non-clathrin endocytosis^58^, tyrosine dephosphorylation of p130^Cas^, actin cytoskeleton rearrangement^59^, inactivation of Src^60^, activation of phospholipase C-gamma 1 (PLCγ1)^57^ and apoptosis^61^. Differences in binding affinities among EGFR ligands^62^ may also modulate the dimer-oligomer balance, giving rise to differential biological consequences downstream.

## Methods

### Cell culture

All reagents unless otherwise stated were from Invitrogen, UK. CHO cells expressing wild-type EGFR or an EGFR mutant (R647C/V650C-EGFR, ΔC-EGFR, C'698-EGFR and C'973-EGFR) under an inducible Tet-ON promoter were a gift from Prof Linda Pike (Washington University). Cells were grown in 5% CO_2_ in air at 37 °C in phenol-red-free DMEM supplemented with 10% (v/v) fetal bovine serum, 2 mM glutamine, 1% penicillin-streptomycin, 100 μg ml^−1^ hygromycin and 100 μg ml^−1^ geneticin. CHO cells with stably transfected EGFR-eGFP were a gift from Prof. Donna Arndt-Jovin (Max Planck Institute for Biophysical Chemistry). All cells used were regularly tested for mycoplasma contamination.

### Fluorophore localization imaging with photobleaching (FLImP)

The method was first described in ref. 15. Briefly 1 × 10^5^ cells were seeded on 1% BSA-coated Piranha cleaned 35 mm no. 1.5 (high tolerance) glass-bottomed dishes (MatTek Corporation, USA) in 2 ml of media plus 50 ng ml^−1^ of doxycycline hyclate (Sigma), resulting in expression of ∼10^5^ receptors per cell^3^. After 48 h the medium was changed to 0.1% serum plus 50 ng ml^−1^ doxycycline for 2 h. CHO cells were rinsed and cooled to 4 °C for 10 min and then labelled with 4 nM EGF-CF640R or Affibody-CF640R for 1 h at 4 °C. The N-terminus of EGF was labelled at a 1:1 ratio by Cambridge Research Biochemicals (Cleveland, UK). The EGFR Affibody was labelled at a 1:1 ratio at its single cysteine residue. For the EGF concentration curve EGF-CF640R was mixed with unlabelled EGF to the required concentration. Cells were rinsed and fixed with 3% paraformaldehyde plus 0.5% glutaraldehyde for 15 min at 4 °C, then 15 min at room temperature. If required, cells were pre-treated for 1 h on ice at 4 °C with 200 nM 9G8 nanobody^4^ dissolved in PBS or for 30 min at 37 °C in 10 mM MβCD. We used an Axiovert 200 M microscope with TIRF illuminator (Zeiss, UK), with a × 100 oil-immersion objective (α-Plan-Fluar, NA=1.45; Zeiss, UK) and an EMCCD (iXon X3; Andor, UK). The microscope is also equipped with a wrap-around incubator (Pecon XL S1). Samples were illuminated using a VortranStradus 638 nm diode laser (Laser Technology, Inc., USA) or a fibre-coupled laser combiner (Andor) with a 100 mW 640 nm diode laser (Cube, Coherent). Images were collected every 0.28 s. For each experiment, ∼120,000 single-particle image spots were obtained from at least 750 cells and at least three biological repeats. Empirical posterior FLImP distributions were then obtained based on discrete EGF or Affibody separation measurements that had confidence intervals of less than 6–7 nm. A more detailed description of the FLImP method can be found in ref. 15, and the theoretical basis of the method is described in Supplementary Methods.

### Characterisation of EGF and affibody binding

CHO cells expressing wild-type EGFR were seeded in 2 ml of media with 250 ng ml^−1^ of doxycycline (∼4 × 10^5^ receptors per cell) and labelled with the required concentration of either EGF- or Affibody-Alexa 488 for 1 h at 4 °C on ice. Cells were rinsed and fixed as described above. Twenty confocal images of equatorial regions of the cells were collected per concentration from three replicates. We used a Leica TCS SP8 microscope with a (NA=1.4; Leica) and a Leica HyD hybrid detector. Samples were illuminated with 488-nm light taken from an NKT Extreme supercontinuum light source. The pixel size of the confocal images was 75 × 75 nm. The fluorescence intensity values of pixels contributing to cell membranes were extracted to create a frequency distribution of membrane pixel intensities for each concentration of EGF- and Affibody-Alexa 488. The median intensity value was plotted as a function of concentration, with error bars representing the upper and lower quartiles of the distribution. To test the effect of binding the 9G8 nanobody to EGFR on the subsequent binding of EGF- and Affibody-Alexa 488, CHO cells were pre-treated with 200 nM 9G8. Frequency distributions of membrane pixel intensities were obtained as above and compared with the distributions derived from 200 nM EGF- or Affibody-Alexa 488 without 9G8.

### Modelling distances between dye pairs

To determine the expected distances between dye pairs in EGFR dimers and tetramers, taking into account the length of the linker and dimensions of the dye molecule, we used a geometric AV algorithm, originally developed for characterizing FRET experiments^18^. Although the structure of CF640R is proprietary, the manufacturer provided us with information on the linker and dye structure that has enabled us to determine its dimensions (Biotium, personal communication). The software for modelling is available on the authors' website (http://www.mpc.hhu.de/en/software/fps.html). A PDB file of the structure of either dimer (1ivo.pdb^7^) or tetramer (model described in the main text, Fig. 2d) was loaded into the ‘AV simulation' software. For each of the fluorescent labels, the attachment atom was specified together with dye and linker dimensions, using a three radius model for the dye. The software outputs both a mean dye position and an AV cloud describing the area accessible to the fluorophore. Average distances between dyes were calculated using the ‘FPSgui' software, giving dye to dye distances of 12.5±0.3 nm for the dimer, and 19.6±0.5 nm for the tetramer. The models are shown in Supplementary Fig. 2b,c.

### FRET distance of closest approach (DOCA)

Uncoated 35 mm no. 1.5 glass-bottomed dishes were seeded with 1 × 10^5^ CHO cells expressing wild-type EGFR in 2 ml of media with 250 ng ml^−1^ of doxycycline (∼4 × 10^5^ receptors per cell). After 2 days, the medium was changed to 0.1% serum with the same concentration of doxycycline for 2 h. Samples were labelled with 5 μM C18 DiD for 10 min at 37 °C, followed by the required mixture of EGF-Alexa 488 and unlabelled EGF at 4 °C on ice for 1 h. Samples were fixed with 3% paraformaldehyde as described above.

Images were collected at room temperature using a Nikon Eclipse C1 confocal microscope with time-correlated single-photon counting electronics (SPC830, Becker-Hickl GmbH) using a supercontinuum light source (Fianium SC450-4; 40 MHz repetition rate). For each field of view, a TCSPC image of Alexa 488 was obtained using 488 nm excitation and fluorescence detection between 505 and 530 nm using a fast photomultiplier tube (PMC-100; Becker-Hickl GmbH). Corresponding confocal intensity images of Vybrant DiD (DOCA) were collected with 640 nm excitation and detection between 670 and 720 nm.

Fluorescent intensity decays were best-fitted to a single exponential decay model, where acceptor was absent and to a bi-exponential model when both donor and acceptor were present using SPCImage FLIM analysis software (Becker-Hickl GmbH). Donor lifetimes for FRET efficiency calculations were obtained by taking the mean of the distribution of the fluorescence lifetimes of pixels. The occurrence of FRET results in a decrease in the fluorescence lifetime of the donor *τ*_D_ in cells loaded with acceptor *τ*_DA_. The FRET efficiency (*E*_FRET_) was calculated from fluorescence lifetime data using the following formula:


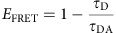


For each cell in the FLIM images, regions of interest were drawn to isolate the membrane and the mean lifetime from these pixels was used to calculate a mean FRET efficiency. The same region of interest was then applied to the corresponding image of of Vybrant DiD labelling to determine the corresponding mean acceptor intensity for that cell. The mean acceptor intensity was converted to a density with units of acceptors per R_0_^2^ using a calibration factor previously determined from samples with a known DOCA^28^. A model obtained from Monte Carlo simulations of a single donor at different distances above a plane of acceptors was fitted to each data set to estimate the ensemble averaged DOCA for that experiment.

### Western blots

CHO cells were seeded in 10 cm plates at the density of 6 × 10^5^ cells per dish with 250 ng ml^−1^ doxycycline hyclate (Sigma). Forty-eight hours later, cells were washed 2 × with ice-cold PBS, then chilled for 10 min on ice and incubated on ice with ice-cold EGF for 1 h at 4 °C using 3 ml solution per dish, then wash 2 × with ice-cold PBS. Cells were scraped into PBS + inhibitors (phosphatases and proteases) and spun down. Cells were lysed in 10 × volume of M-PER + 100 mM NaF + 1 mM Na3VO4 + 1% protease inhibitors + 150 mM NaCl + 1mM EDTA at pH 8 and incubated for 10 min at room temperature. Cells were cleared by centrifugation and total protein measured. Sample buffer was added to 1 × final concentration. Samples were run in parallel on 1.0-mm thick (or 1.5-mm thick for EGF response curve), 3–8% Tris-Acetate NuPAGE gels (Invitrogen) with HiMark Prestained HMW and Novex Sharp Prestained protein standards (Invitrogen) using an XCell apparatus (Invitrogen). Proteins were blotted using an iBlot system (Invitrogen) on polyvinylidene difluoride membranes, blocked for 1 h at 4 °C with 5% BSA in TBS+0.1% Tween and probed overnight with mouse anti-phosphotyrosine 4G10, 1:1,000 (05-321), rabbit anti-EGFR pY1173 1:1,000 (04-341) (both Upstate (Millipore)) or mouse anti-EGFR pY992 antibody [EM-12] (ab81440) (Abcam). Gels were probed with secondary anti-mouse or anti-rabbit horseradish peroxidase antibody (Jackson ImmunoResearch) and incubated with Supersignal West Pico Chemiluminescent Substrate solution (Pierce) for 5 min, then imaged with a BioRad ChemiDoc MP system imager. Each blot was stripped with 25 ml stripping buffer (2% SDS, 0.75% β-mercaptoethanol, 62.5 mM Tris HCl pH 6.7) for 50 min at 60 °C, and re-probed with an anti-EGFR cocktail composed of anti-EGFR D38B1, 1:2,000 (#4264) (Cell Signalling Technologies), anti-EGFR N-Terminal polyclonal, 1:2,000 (ab137660) (Abcam) and anti-EGFR polyclonal 10005, 1:2,000(sc-03) (Santa Cruz Biotechnology), all derived from rabbit. Anti-rabbit horseradish peroxidase (Jackson ImmunoResearch) was used for all blots and images were acquired as above. Uncropped western blots are shown in Supplementary Fig. 14.

### Tracking of EGFR complexes on CHO cells

EGFR expression was induced with 50 ng ml^−1^ doxycycline and if needed, on the following day, cells were transfected with 1 μg of PLCδ1-PH-eGFP pCDNA plasmid (gift from Prof Banafshe Larijani, Universidad del Pais Vasco) using Fugene HD (Roche) as a carrier. Expression was allowed to proceed for 24 h. Before imaging, cells were starved for 2 h at 37 °C in 0.1% serum supplemented with 50 ng ml^−1^ doxycycline. Cells were rinsed twice with 0.1% serum without doxycycline pre-heated at 37 °C. Labelling with Affibody-Alexa 488 was carried out for 10 min at 37 °C. Cells were rinsed twice with low serum medium without doxycycline pre-heated at 37 °C and promptly imaged as described in Supplementary Fig. 10.

### Mean squared displacement calculations

From single-particle tracks, MSD curves were calculated as MSD(*ΔT*)=<|**r**_**i**_(*T+ΔT*)−**r**_**i**_(*T*)|^2^>, where |**r**_**i**_(*T+ΔT*)−**r**_**i**_(*T*)| is the displacement between position of track *i* at time *T* and time *T*+*ΔT* and the average value is overall pairs of points separated by *ΔT* in each track. The average instantaneous diffusion coefficient (*D*) for these tracks was calculated by fitting a straight line to the first two points of the MSD curve then calculating *D* directly from the gradient *m* of the fit, *D*=*m*/4. The errors in the MSD curve were calculated by repeating the MSD curve calculation 200 times, each time on a different synthetic data set created by randomly resampling with replacement the time points within each track, the tracks present within each data set, and the data sets present (bootstrap resampling^63^). The error in *D*, σ(*D*), is calculated from the s.d. of the *D* fits obtained from each bootstrap-resampled MSD curve.

### Molecular dynamics simulations of the EGFR tetramer

Each monomer in the EGFR tetramer model contained residues 1–995, including 40 residues that are part of the C-terminal tail. The simulation systems also included two EGF molecules bound to the tetrameric extracellular domains of EGFR. At the beginning of the simulations, the two asymmetric kinase dimers were not in close contact with one another (Supplementary Fig. 5d). Two separate systems of the EGFR tetramer were set up and simulated. One system (Simulation 1, Fig. 2c) of 24.3 × 24.3 × 21.5 nm^3^ in dimensions contained total 1,151,739 atoms and was simulated up to 40.6 μs. A smaller but otherwise largely similar system (19.6 × 13.6 × 23.0 nm^3^ in dimensions, containing 579,306 atoms) was simulated up to 10.6 μs (Simulation 2, Supplementary Fig. 5d). The simulation system of the face-to-face EGFR extracellular dimer built based on HER3 crystal dimer had dimensions of 17.0 × 17.0 × 17.0 nm^3^ and contained 472,072 atoms in total; this simulation was up to 5.98 μs in length (Simulation 3). Each EGFR monomer in system contained residues 1–614. To mimic the charge distribution in the cellular membrane^64^, the model membrane consisted of 100% 1-palmitoyl-2-oleoylphosphatidylcholine (POPC) lipids in the extracellular leaflet and 70% POPC plus 30% 1-palmitoyl-2-oleoylphosphatidylserine (POPS) lipids in the intracellular leaflet of the bilayer. The distance between the EGF N-terminus and the membrane (as determined by the distance from the N-terminus to the plane through the phosphates of the extracellular lipid layer) was computed in a manner consistent with the FRET measurements, but the size of the fluorophore was not taken into account. The simulations were performed on a special-purpose supercomputer, Anton 2 (ref. 65), using the Amber ff99SB-ILDN (ref. 66) force field for proteins, the CHARMM C27 force field^67^ for lipids, and TIP3P (ref. 68) for water. The force field for palmitic acid in the simulations of palmitoylated R647C/V650C mutant was from Forcefield_PTM (ref. 69). The simulated systems were solvated in water with 0.15 M NaCl, with residue protonation states corresponding to pH 7. Additional Na^+^ ions were included to neutralize the net charges of the proteins (−3 for the extracellular domains of each EGFR, −4 for each EGF ligand) and the POPS lipids. As an equilibration stage, the protein backbone atoms were first restrained to their initial positions using a harmonic potential with a force constant of 1 kcal mol^−1^ Å^−2^. The force constant was linearly scaled down to zero over at least 50 ns. Simulations were performed in the constant number (N), pressure (P), and temperature (T) (NPT) ensemble with *T*=310 K and *P*=1 bar using a variant^70^ of the Nosé–Hoover^71^ and the Martyna–Tobias–Klein algorithm^72^. Water molecules and all bond lengths to hydrogen atoms were constrained using M-SHAKE^73^. The simulation time step was 1 fs in the equilibration stage and 2 fs in production simulations; the r-RESPA integration method was used, with long-range electrostatics evaluated every three time steps electrostatic forces were calculated in Simulation 1 using the u-series method, a recently developed Ewald-like method^65^, with a 1.37 nm cutoff for the electrostatic pairwise summation; a 0.9 nm cutoff for the van der Waals calculations. The electrostatic forces in Simulations 2 and 3, which were performed earlier, were calculated using the earlier developed Gaussian split Ewald method^74^ with a 1.465 nm for the electrostatic pairwise summation, and a 1.05 nm cutoff for the van der Waals calculations. All three simulations used a 64 × 64 × 64 mesh for the distant electrostatic calculations.

### Data availability

All relevant data are available from the authors on request and/or are included with the manuscript (as figure source data or Supplementary Information Files).

## Additional information

**How to cite this article:** Needham, S. R. *et al*. EGFR oligomerization organizes kinase-active dimers into competent signalling platforms. *Nat. Commun.* **7,** 13307 doi: 10.1038/ncomms13307 (2016).

**Publisher's note:** Springer Nature remains neutral with regard to jurisdictional claims in published maps and institutional affiliations.

## Supplementary Material

Supplementary Information Supplementary Figures 1-14, Supplementary Methods and Supplementary References.

Supplementary Movie 1 Single particle tracking of EGFR on live CHO cells at 37°C. CHO cells stably expressing 10^5^ wild type EGFR/cell were treated with a 4 nM CF640R fluorophore-conjugated Affibody solution at 37°C and tracked as previously described^7^. The multiple steps during photobleaching shown by individual spots indicate that receptors form high order oligomers prior to the EGF stimulus.

Supplementary Movie 2 Single particle tracking of EGFR on live CHO cells at 37°C. CHO cells stably expressing 10^5^ wild type EGFR/cell were treated with a 4 nM CF640R fluorophore-conjugated EGF solution at 37°C, and tracked as previously described^7^. The multiple steps photobleaching shown by individual spots indicate that receptors also move in clusters larger than dimers after the EGF stimulus.

Dataset 1 The 3-dimension coordinates of the tetramer model of full-length EGFR up to residue 995 and the membrane lipids are prepared in PDB format. The coordinates are taken from the end of the simulation of the model at 40 μs.
