# Supplementary material for: EGFR oligomerization organizes kinase-active dimers into competent signalling platforms
Source: Nat Commun. 2016 Oct 31;7:13307. doi: 10.1038/ncomms13307 (PMC5095584; doi:10.1038/ncomms13307)
Supplement: Supplementary Information — Supplementary Figures 1-14, Supplementary Methods and Supplementary References. [file ncomms13307-s1.pdf]

SUPPLEMENTARY FIGURES

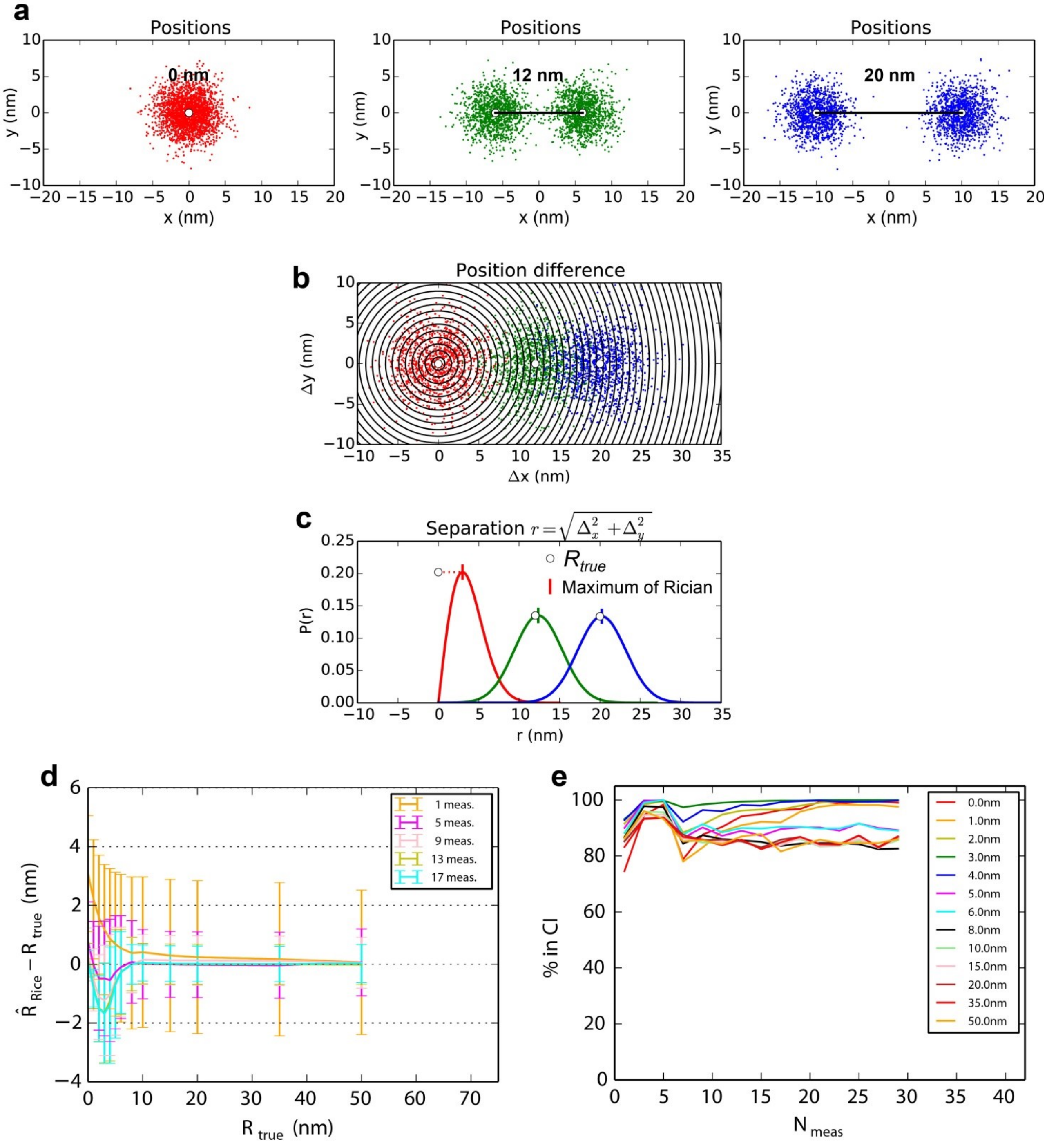

Supplementary Figure 1| Origin of the Rice distribution asymmetry and the bias of distances smaller than measurement resolution. (a) Model of data for three separations (0, 12, and 20nm). Because of particle localization errors (for our measurements around 2-4 nm) an individual pairwise separation will appear as a distribution with a typical combined localization error of ~4-6nm[1]. For the two longer distances, the separations are clearly resolved while they overlap for the zero separation model. **(b)** 2D illustration of lateral separations measured from the data in **(a)**. The black circles are contours of constant separation. The distributions of separations (shown in **(c)**) are calculated by integrating around each annulus. For each separation r_i, the annulus that contains the highest number

of dots is equivalent to the maximum of the Rician distribution. For the two separations > the localization error (12 and 20nm), the maximum of the Rician distribution is almost identical to the true separation (R_{true}). For separations < the localization error, like r = 0nm, there is a significant deviation between the true separation and the Rician maximum (red dotted line). This effect depends on the size of the localization error and is further explored in **(d)** and **(e)**, which show confidence interval testing for the Rician approximation to the FLImP distribution $H(r)$. Monte Carlo simulations of $H(r)$ were calculated for N_{comp} = 1, for N_{meas} in the range 1-30, where N_{comp} is the number of components and N_{meas} the number of FLImP empirical posterior measurements, with simulated FLImP data representative of those in this paper. 500 simulations were performed for each combination of N_{meas} and R_{true}. **(d)** Shows the mean and standard deviation between the best fit Rice separation and R_{true} as a function of R_{true} and N_{meas} revealing biases from the properties of the Rice distribution for small separations. **e)** Shows the proportion of those simulations with R_{true} within the confidence interval ($\hat{R}_{true,j,peak,low}$, $\hat{R}_{true,j,peak,high}$) (**Supplementary Methods**). Because of the errors and biases in the FLImP analysis when the separation approaches the localization error, FLImP separations are only given for distances in which the Rician best fit value is > 6nm.

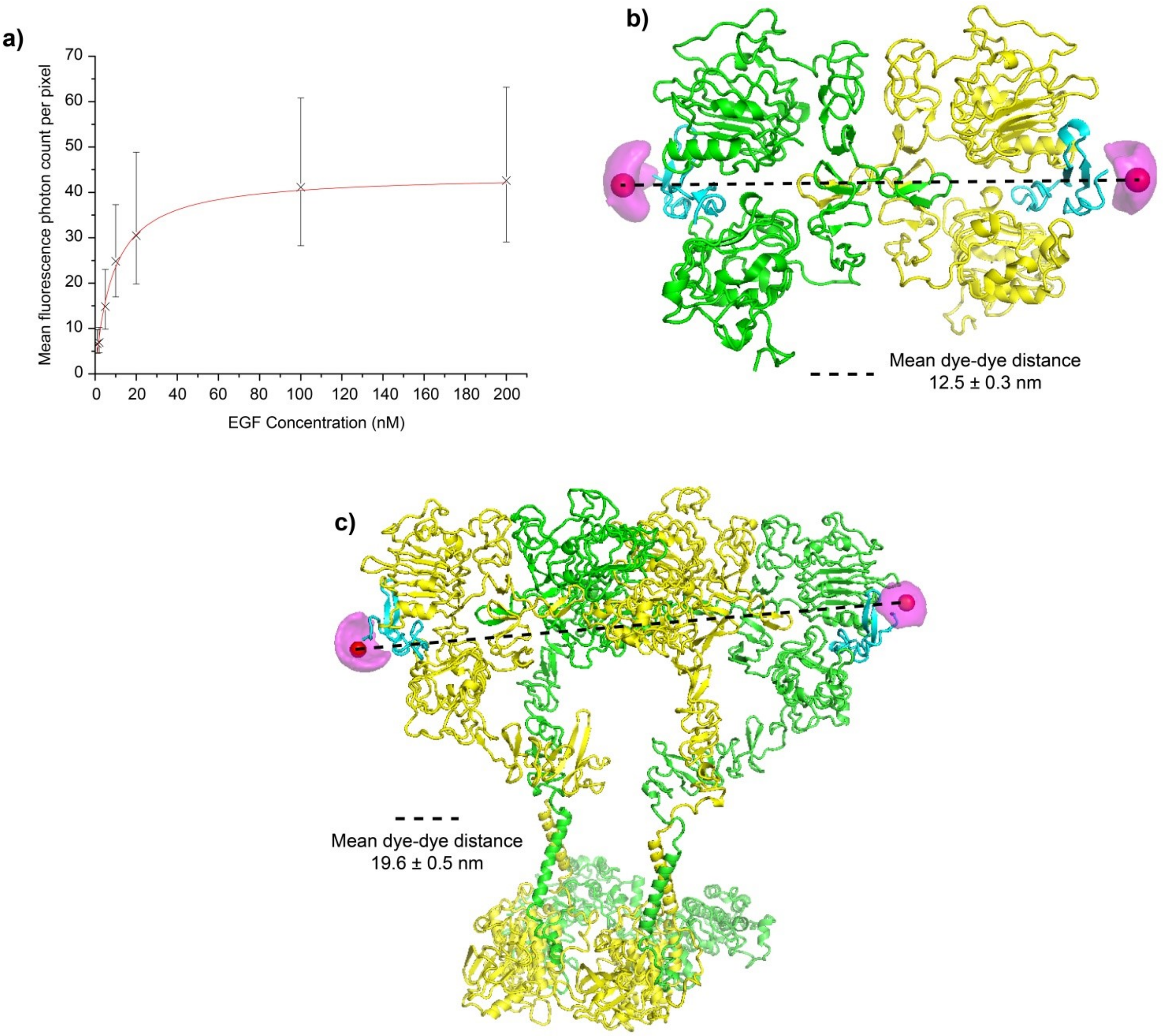

Supplementary Figure 2| Occupancy of EGFR ligand binding sites as a function of EGF and Affibody concentrations and calculation of mean distances between fluorescent dyes. (**a)** The median fluorescence intensity of EGF-Alexa 488 at the membrane of CHO cells that express ~$4x10^5$ copies of wild type EGFR per cell, measured by confocal microscopy for increasing concentrations of fluorescent EGF. Cultured cells were exposed to increasing concentrations of EGF-Alexa 488 until the fluorescence reached a near plateau. The level at near fluorescence saturation values (200 nM EGF) were >10x larger than the values at 4 nM. Experiments were repeated in triplicate (Error bars are standard deviation). (**b)** Calculation of mean distance between fluorophores for EGFR labeled with CF640R at the N-terminus, using the dimer structure 1IVO.pdb[2]. EGFR are shown in green and yellow, bound EGF in cyan. The red spheres indicate the mean position of the dyes, and the magenta surfaces the volume accessible to the dye, calculated using a geometric accessible volume algorithm[3], as described in **Methods**. **(c)** As **(b)** but calculated for labeled EGF in the full length tetramer model described in the main text (**Fig. 2d**).

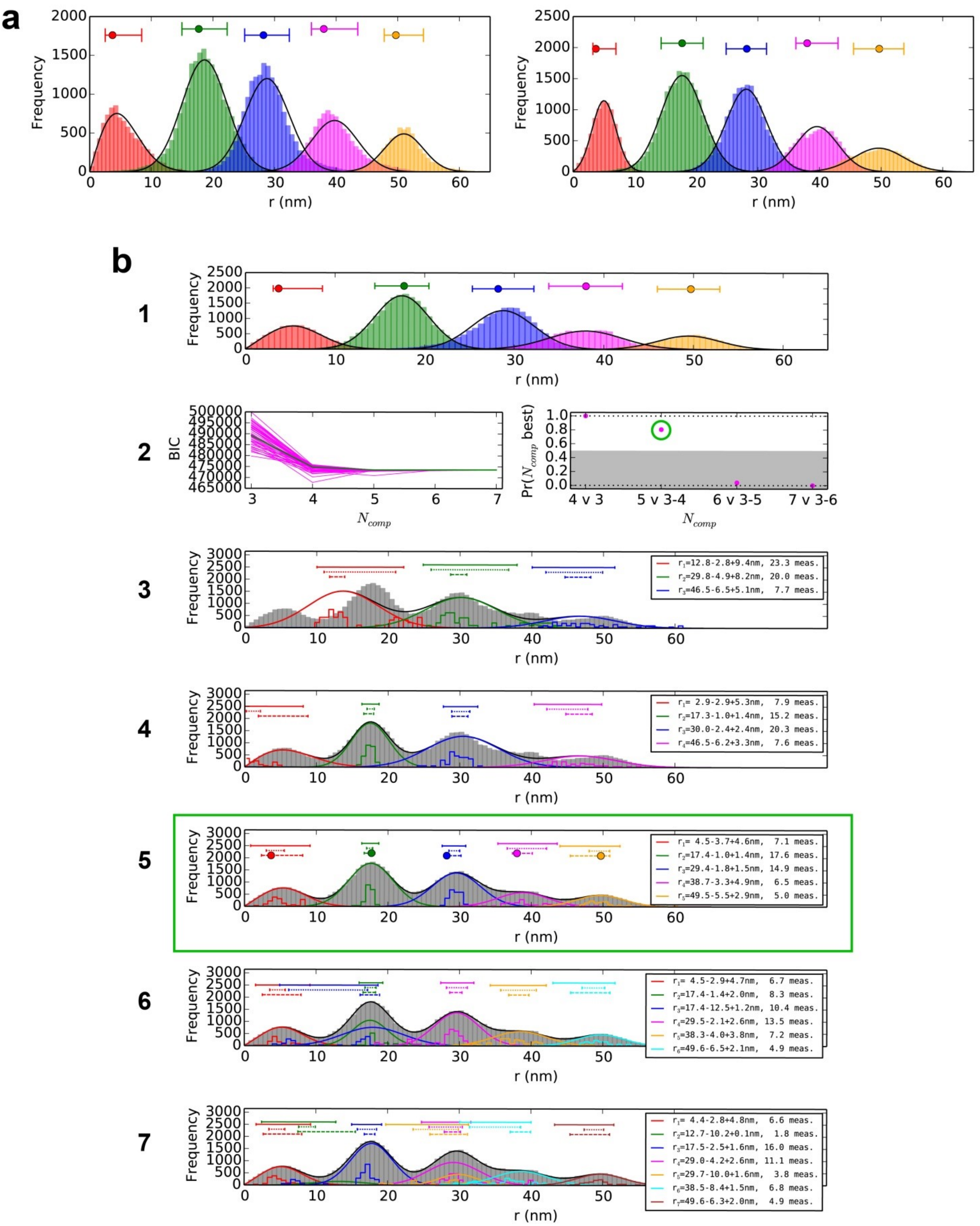

Supplementary Figure 3| Model selection using a Bayesian Information criterion. (a) Monte Carlo simulations of FLImP distributions with data quality representative of that in this paper, showing two simulations for the same ground truth. Annotation shows the ground truth separations (circle), Rician distributions separately fitted to each distance (black curves) and the confidence intervals from the fits calculated as described. There are deviations from a perfect Rician distribution,

whose effect is accounted for by our determination of confidence intervals (See **Supplementary Methods**). **(b)** Example of the full fitting and model selection for a five separation ground truth. (1) Separate simulated components as in (**a**). (2) Model selection using Bayesian information criterion (BIC). *Left panel*: BIC as a function of the number of components, N_{comp} for the true data (green) and for each of the bootstrap resampled datasets (purple). The purple curve has been shifted vertically to have the same minimum as the green one to make the variation as a function of N_{comp} (which is what matters) clearer. *Right panel*: the proportion of BIC curves for the data/bootstrap resampled data for which each N_{comp} is significantly better (BIC at least 10) than all lower N_{comp}. It is therefore the probability that each N_{comp} is justified over all lower N_{comp}. We chose models in the white region only, i.e. where it was more probable than not that the chosen N_{comp} was justified over simpler models. (Panels 3-7) For each considered number of components, a histogram of the pooled FLImP measurements (or FLImP distribution) (grey), the best fit Rician peak (coloured for each peak), the sum of the best fit Ricians (black line, this is also the best fit to the histogram), the distribution of fitted peak centres for the bootstrap resampled[4] datasets (coloured histogram under each peak), and the confidence intervals ($\hat{R}true,j,peak,low$, $\hat{R}true,j,peak,high$), ($\hat{R}true,j,boot,low$, $\hat{R}true,j,boot,high$) and final combined intervals, ($\hat{R}true,j,low$, $\hat{R}true,j,high$), (dashed, dotted and solid bars respectively). The ground truth separation are also marked (circles). The best solution indicated by BIC (green circle in (2) *Right panel*) is highlighted by a green box in (5).

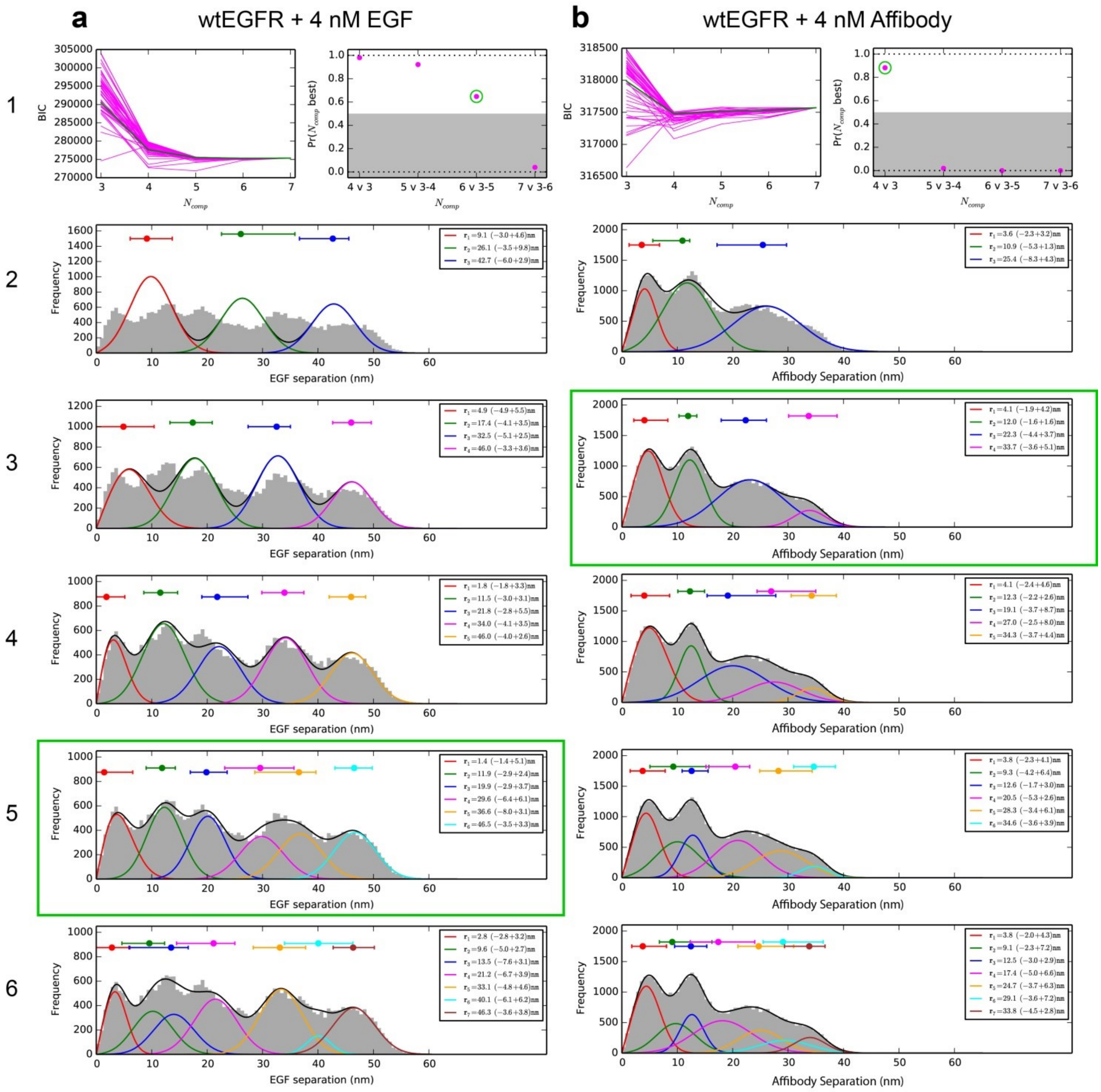

Supplementary Figure 4| FLImP decomposition into its underlying Rician-peak components objectively determined by a Bayesian Information Criterion. (a) Decomposition of the FLImP distribution at 4 nM EGF shown in main text (**Fig.1c**). (1) Bayesian information criterion (BIC) as described in **Supplementary Fig. 3b** and **Supplementary Methods)**. The figure shows that increasing the number of peaks from 6 to 7 is not objectively justified by the data (< 5% of the samples have an improvement in BIC of ~ 10). (2-6) Different fits using increasing numbers of peak components. The model suggested by the BIC calculation (6 underlying peak components) is highlighted by a green box. **(b)** Similar to **(a)** for the 4 nM anti-EGFR Affibody FLImP distribution shown in (**Fig. 7b**) in main text. In this case the BIC calculation shows that the data is best decomposed using 4 underlying peak components.

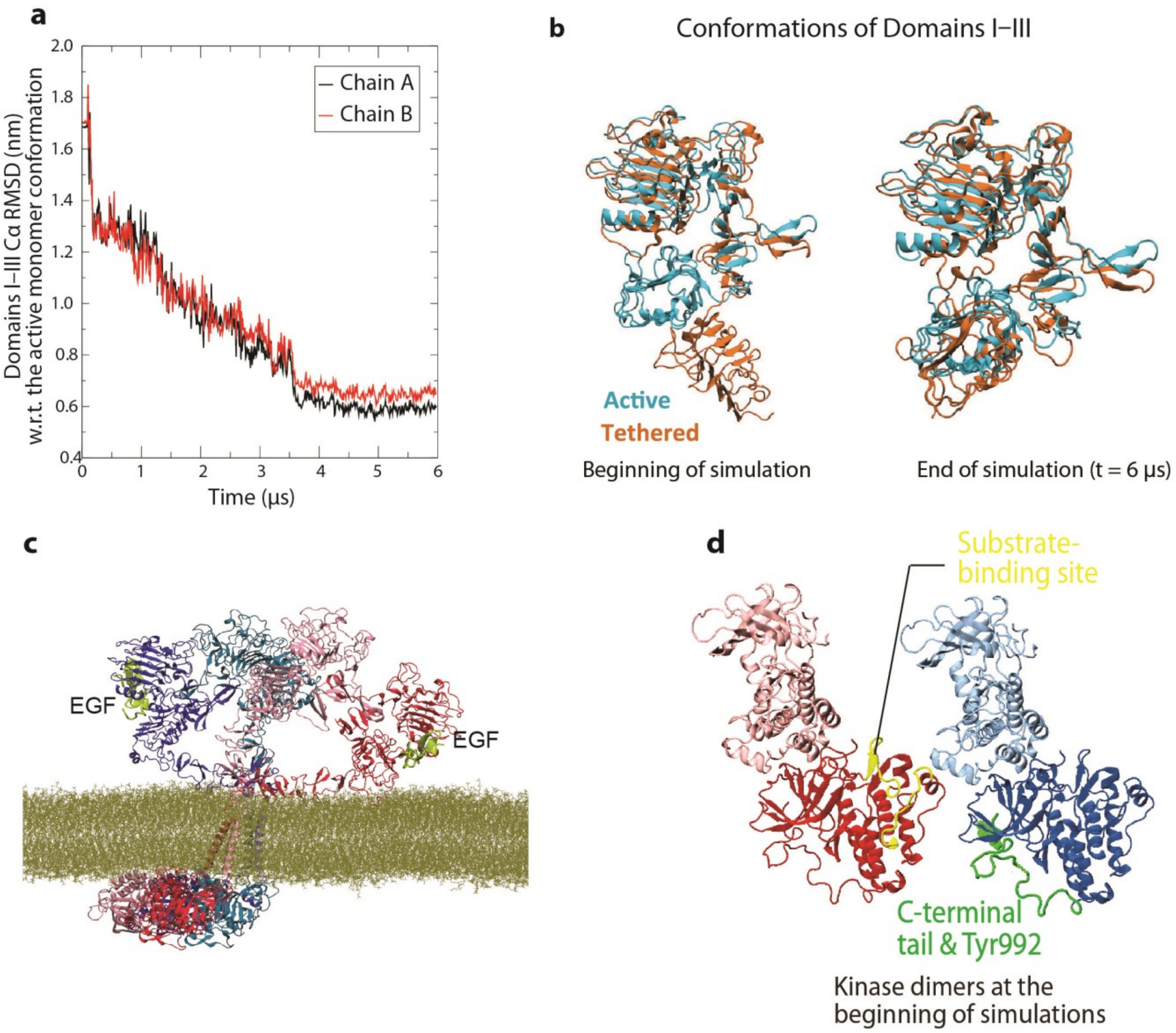

Supplementary Figure 5| Domains I-III depart from the tethered conformation in simulation. **(a)** Root-mean-square-deviation (RMSD) of the domains I–III of each monomer with respect to the tethered conformation in the simulation of the dimer model of EGFR extracellular domains based on the HER3 crystal structure (**Fig. 1a**, step 2). The conformational change of the two monomers is highly concerted. **(b)** The conformations of domains I–III at the beginning and the end of the simulation compared with the conformation of a monomer of the 2-ligand active dimer. **(c)** The extracellular tetramer model in a simulation of over 10μs, in which the distance from one of the two bound EGF ligands to the membrane was particularly short. **(d)** Two asymmetric kinase dimers in the tetramer model at the beginning of the simulations, which were not in close contact with one another.

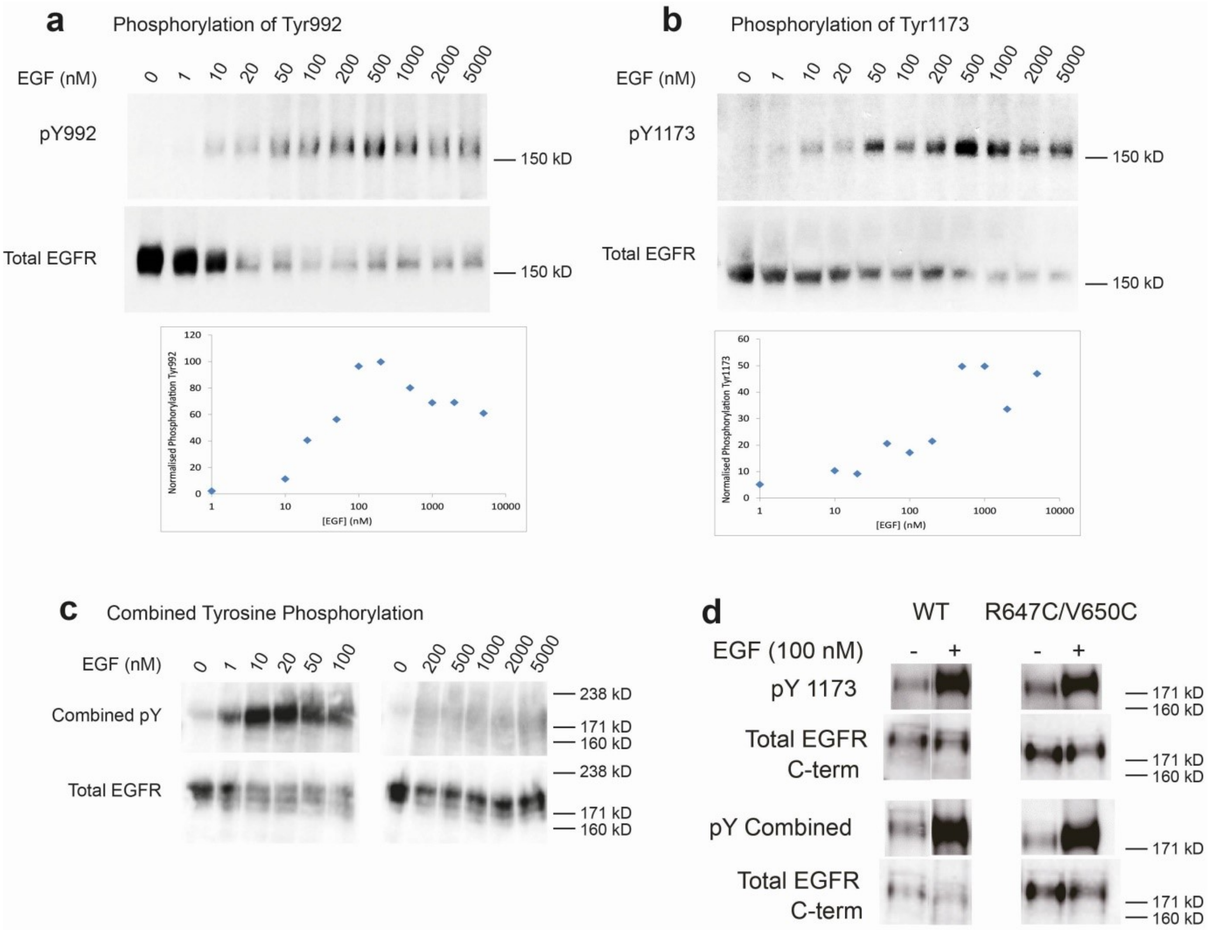

Supplementary Figure 6| Phosphorylation of Tyr992 and Tyr1173 and total EGFR phosphorylation for wild type EGFR and phosphorylation of the R647C/V650C mutant. a) Example of phosphorylation of Tyr992 of EGFR detected with mouse anti-EGFR pY992 antibody EM-12, and **b)** phosphorylation of Tyr1173 detected with rabbit anti-EGFR pY1173 as a function of EGF dose response. Total EGFR was probed with an anti-EGFR cocktail (D38B1, ab137660 and 10005: sc-03). Normalized quantifications for these blots are shown underneath. **(c)** EGFR phosphorylation on CHO cells expressing wild type EGFR detected with pan-phosphorylation 4G10 antibody as a function of EGF dose response. Total EGFR probed as in **(a)** and **(b)**. **(d)** A comparison of the total phosphorylation and Tyr1173 phosphorylation between wild type EGFR and the R647/V650C-EGFR mutant on cells treated with 100 nM EGF.

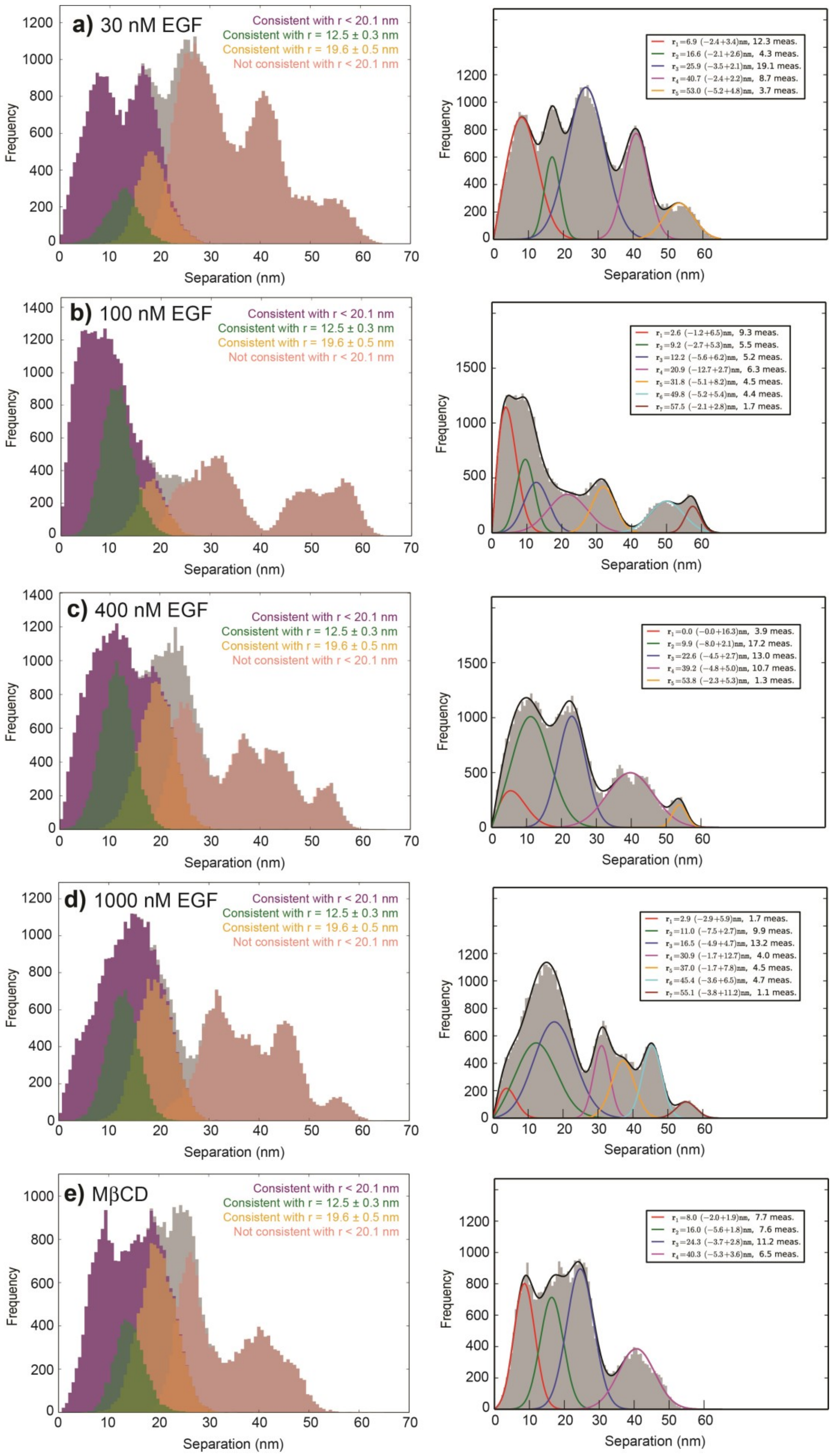

a) 30 nM EGF
Consistent with r < 20.1 nm
Consistent with r = 12.5 ± 0.3 nm
Consistent with r = 19.6 ± 0.5 nm
Not consistent with r < 20.1 nm
Frequency
Separation (nm)
r1=6.9 (−2.4+3.4)nm, 12.3 meas.
r2=16.6 (−2.1+2.6)nm, 4.3 meas.
r3=25.9 (−3.5+2.1)nm, 19.1 meas.
r4=40.7 (−2.4+2.2)nm, 8.7 meas.
r5=53.0 (−5.2+4.8)nm, 3.7 meas.
b) 100 nM EGF
Consistent with r < 20.1 nm
Consistent with r = 12.5 ± 0.3 nm
Consistent with r = 19.6 ± 0.5 nm
Not consistent with r < 20.1 nm
r1=2.6 (−1.2+6.5)nm, 9.3 meas.
r2=9.2 (−2.7+5.3)nm, 5.5 meas.
r3=12.2 (−5.6+6.2)nm, 5.2 meas.
r4=20.9 (−12.7+2.7)nm, 6.3 meas.
r5=31.8 (−5.1+8.2)nm, 4.5 meas.
r6=49.8 (−5.2+5.4)nm, 4.4 meas.
r7=57.5 (−2.1+2.8)nm, 1.7 meas.
c) 400 nM EGF
Consistent with r < 20.1 nm
Consistent with r = 12.5 ± 0.3 nm
Consistent with r = 19.6 ± 0.5 nm
Not consistent with r < 20.1 nm
r1=0.0 (−0.0+16.3)nm, 3.9 meas.
r2=9.9 (−8.0+2.1)nm, 17.2 meas.
r3=22.6 (−4.5+2.7)nm, 13.0 meas.
r4=39.2 (−4.8+5.0)nm, 10.7 meas.
r5=53.8 (−2.3+5.3)nm, 1.3 meas.
d) 1000 nM EGF
Consistent with r < 20.1 nm
Consistent with r = 12.5 ± 0.3 nm
Consistent with r = 19.6 ± 0.5 nm
Not consistent with r < 20.1 nm
r1=2.9 (−2.9+5.9)nm, 1.7 meas.
r2=11.0 (−7.5+2.7)nm, 9.9 meas.
r3=16.5 (−4.9+4.7)nm, 13.2 meas.
r4=30.9 (−1.7+12.7)nm, 4.0 meas.
r5=37.0 (−1.7+7.8)nm, 4.5 meas.
r6=45.4 (−3.6+6.5)nm, 4.7 meas.
r7=55.1 (−3.8+11.2)nm, 1.1 meas.
e) MβCD
Consistent with r < 20.1 nm
Consistent with r = 12.5 ± 0.3 nm
Consistent with r = 19.6 ± 0.5 nm
Not consistent with r < 20.1 nm
r1=8.0 (−2.0+1.9)nm, 7.7 meas.
r2=16.0 (−5.6+1.8)nm, 7.6 meas.
r3=24.3 (−3.7+2.8)nm, 11.2 meas.
r4=40.3 (−5.3+3.6)nm, 6.5 meas.

Supplementary Figure 7| Distributions of pairwise EGF separations in EGFR complexes. The FLImP distribution (grey) of wild-type EGFR expressing CHO cells treated with: **(a)** 30 nM total EGF concentration (8 nM labeled + 22 nM unlabeled); **(b)** 100 nM EGF (10 nM labeled + 90 nM unlabeled); **(c)** 400 nM EGF (20 nm labelled + 380 nM unlabeled); and **(d)** 1 μM EGF (50 nM labeled + 950 nM unlabeled). (*Left column*) The proportions of labeled EGF relative to the unlabeled were chosen to avoid overcrowding of fluorescent spots in single-molecule images[5] and they do not affect the results of the FLImP measurements, which are stochastic in nature. The fraction of FLImP separations whose confidence intervals overlap with the expected 12.5 ± 0.3 dimer interval (green), expected 19.6 ± 0.5 tetramer interval (orange), the dimer/tetramer range (0-20.1 nm) (purple) or not consistent with any of the above ranges (pink) are shown. (*Right column*) Decomposition of FLImP distributions into a number of discrete Rician peaks with errors as specified by the BIC method (see **Supplementary Methods**). The FLImP distributions include the following number of FLImP measurements: **(a)** 48, **(b)** 37; **(c)** 46; **(d)** 39; and **(e)** 33, and with confidence intervals < 6 or 6.5 nm.

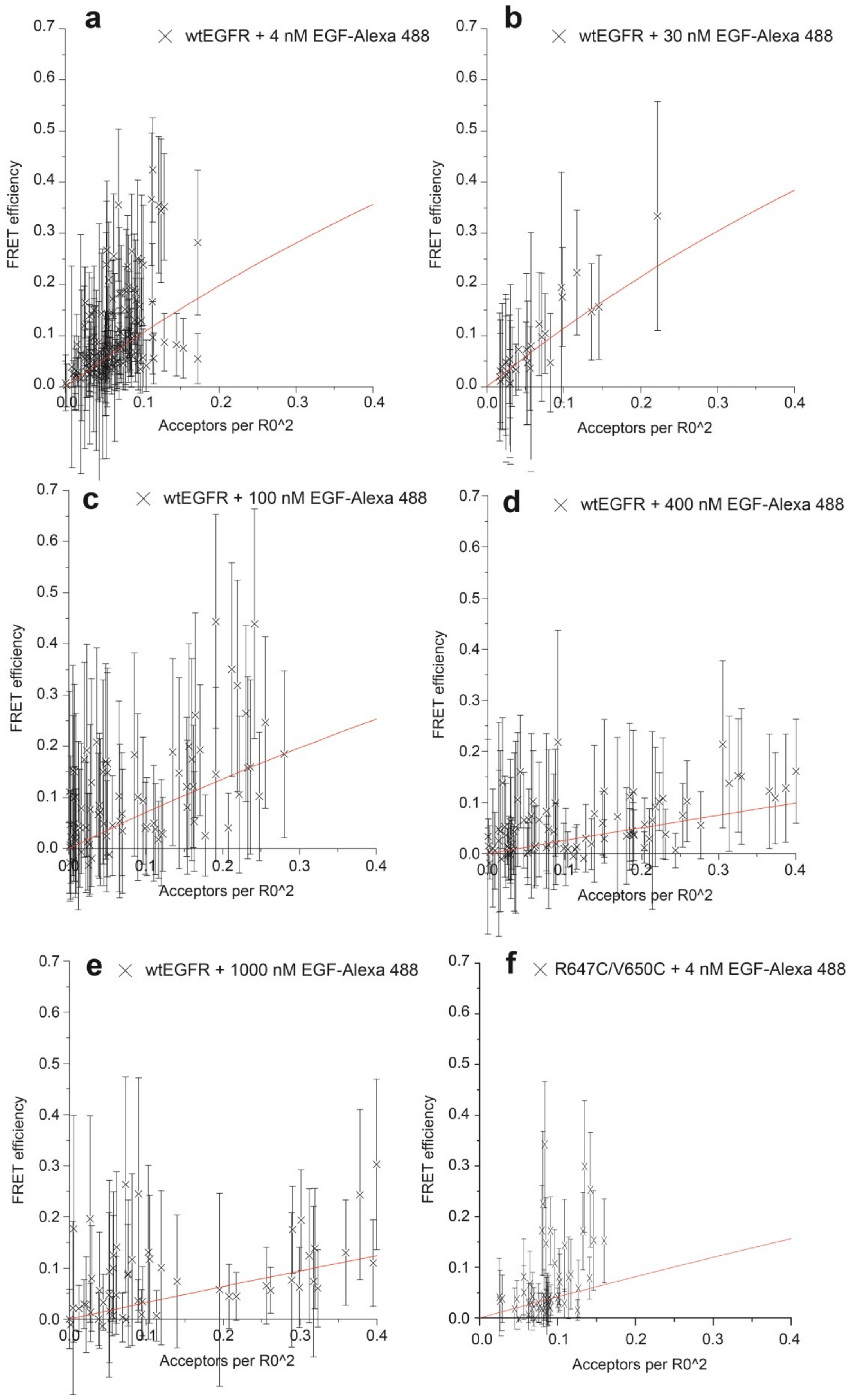
a
wtEGFR + 4 nM EGF-Alexa 488
b
wtEGFR + 30 nM EGF-Alexa 488
c
wtEGFR + 100 nM EGF-Alexa 488
d
wtEGFR + 400 nM EGF-Alexa 488
e
wtEGFR + 1000 nM EGF-Alexa 488
f
R647C/V650C + 4 nM EGF-Alexa 488
FRET efficiency
Acceptors per R0^2
0.0
0.1
0.2
0.3
0.4
0.5
0.6
0.7

Supplementary Figure 8| Determination of the distance of closest approach (DOCA) of EGF ligands to the cell surface. Plots of FRET efficiency as a function of acceptor density measured in acceptor (DiD)-loaded CHO cells expressing wild-type EGFR at a level of ~4 x10^5 receptors per cell, using a previously described method[6]. The receptors are labelled with EGF-Alexa 488 (10% of total EGF is labeled with Alexa 488). **(a)** FRET efficiency at 4 nM EGF **(b)** FRET efficiency at 30 nM EGF **(c)** FRET efficiency at 100 nM EGF **(d)** FRET efficiency at 400 nM EGF **(e)** FRET efficiency at 1 μM EGF **(f)** Similar to **a)** on CHO cells expressing the R647/V650C-EGFR mutant treated with 4 nM EGF. The best fits of Monte Carlo simulation results to the data are shown[6]. The errors from uncertainty in the Monte Carlo model fitting were typically 10-20% of the calculated distance values as previously described, determined by bootstrapping with 1000 bootstrap datasets resampled with replacement.

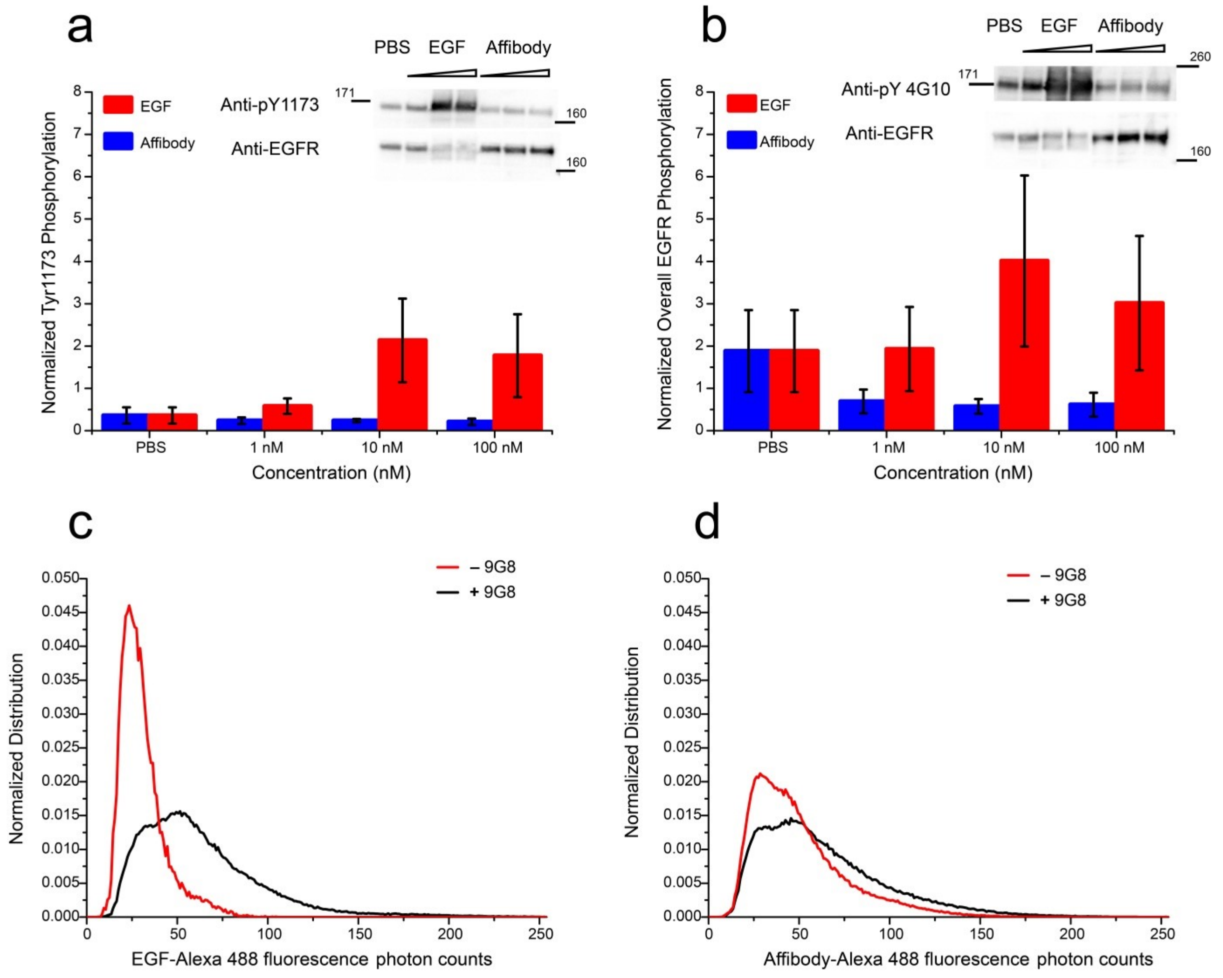

Supplementary Figure 9| The Affibody does not activate EGFR, and promoting the tethered conformation with the 9G8 nanobody has little effect on Affibody binding. Western blot results using **(a)** pTyr1173-specific antibody or **(b)** using the pan-phosphotyrosine antibody 4G10 and an antibody against the EGFR N-terminal tail from CHO cells (expressing wild-type EGFR) treated with 1, 10, or 100 nM of Affibody or EGF for 1 hour at 4°C. The phosphorylation signals are normalized to total EGFR signal and the mean and standard deviation of three replicates has been plotted. Inset is an image of one of these replicates. **(c)** The binding of 200 nM EGF-Alexa 488 to EGFR (red), assessed by the distribution of fluorescence photon counts per pixel of membrane (obtained with confocal microscopy). The photon counts are greatly reduced when cells are exposed to a saturating concentration (200 nM) of the 9G8 nanobody before EGF binding (black). **(d)** In contrast, when the binding of 200 nM Affibody-Alexa 488 is assessed (red), only a small change in the distribution is caused by pre-treatment with 200 nM 9G8 (black).

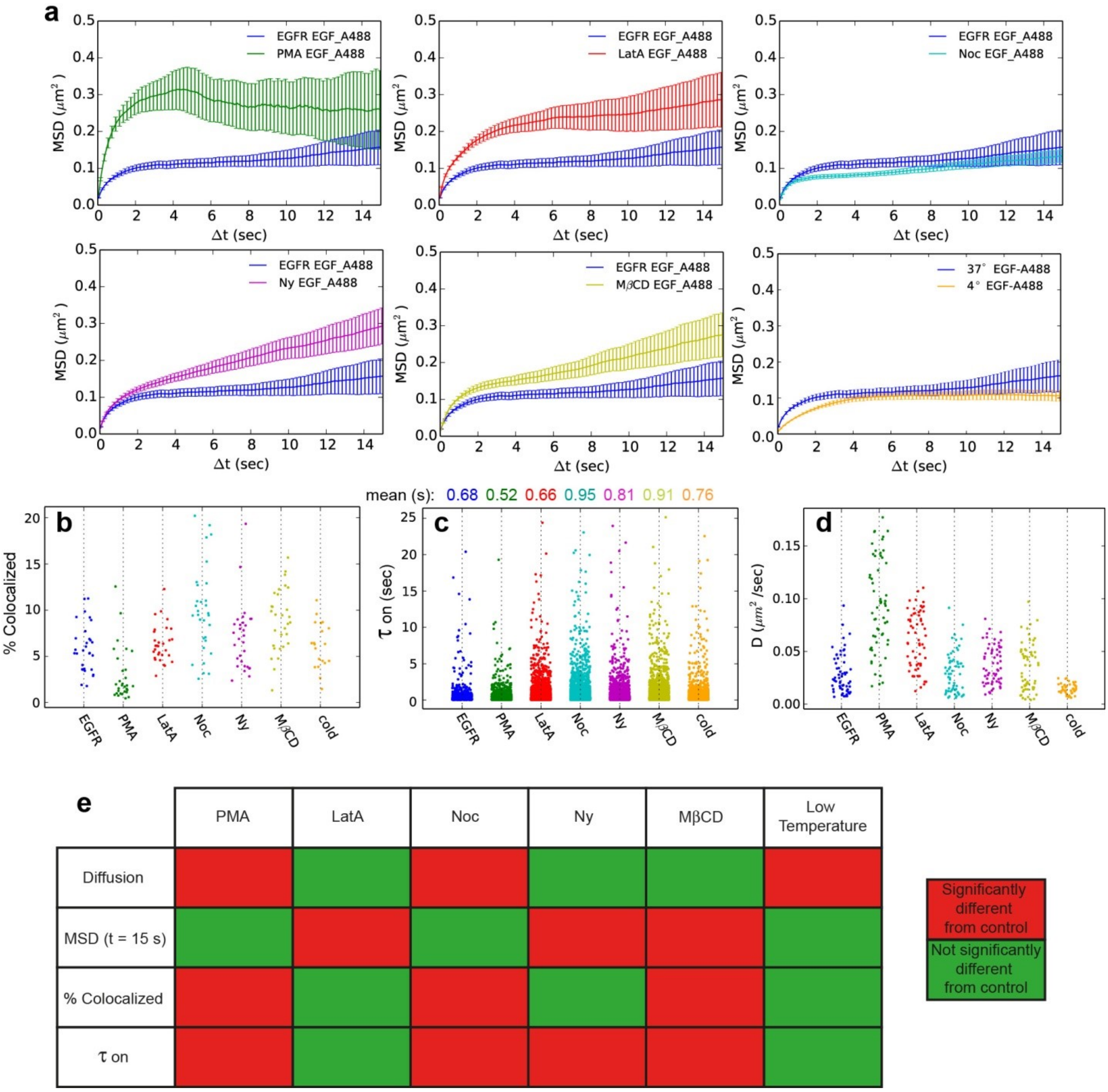

Supplementary Figure 10: Exploring artifacts from low temperature. For single-molecule tracking wild-type and mutant EGFR expressing CHO cell lines were seeded at a density of 105 cells on 1% BSA-coated Piranha cleaned 35 mm no. 1.5 (high tolerance) glass-bottomed dishes in 2 ml of media plus 50 ng/ml of doxycycline hyclate, resulting in expression of ~105 receptors/cells. Prior to imaging, cells were starved for 2 h at 37 °C in 0.1% serum medium supplemented with 50 ng/ml doxycycline. After starvation, cells were rinsed twice with 0.1% serum medium without doxycycline pre-heated at 37°C. An 8 nM solution of 4 nM EGF-CF640R and 4nM EGFR-Alexa 488 was added to cell culture dishes and cells were promptly imaged without rinsing steps, in order to minimise internalisation during labelling and imaging. Two-color TIRF images of the basolateral surfaces of cells were chromatically separated by a beam splitter and registered using custom made software to map the relative positions of the probes over the time course of data acquisition. Single particle tracks were extracted as previously described[7].

[7]. **(a)** MSD-curves for tracks of EGF-bound EGFR with bootstrap-estimated errors (vertical line). The MSD of EGFR treated with 4 nM EGF at 37°C (blue) is shown for comparison. Other colours show MSDs for EGFR on cells treated at 37°C with 100mM phorbol myristate acetate (PMA) (green), 10µM Latrunculin A (Sigma) to disrupt the cytoskeleton (red), 10µM Nocodazole (AppliChem) to disrupt microtubules (pale blue), 5 µg/ml Nystatin (Sigma) (purple), 10 mM Methyl-β-cyclodextrin (Sigma) (pale green), and 4nM EGF at 4°C (orange). MSD were derived as described[7]. **(b)** For all treatments in **(a)**, fractions of tracks in which the features in two tracks in different colour channels spent at least 3 frames (150 ms) moving together while remaining within a distance of each other of less than one pixel. The data are shown with horizontal spread to separate data points within each condition. Coincidental colocalization statistics were accounted for. To reduce the impact of localization error on these results a temporal Gaussian smoothing filter of FWHM 4 frames (200 ms) was applied to the position traces before the colocalisation analyses. **(c)** Duration of the colocalisation events from the time a feature in one channel begins to move together within a pixel of a feature in the other channel until then they move apart. **(d)** Instantaneous diffusion rate (*D*) estimated from the MSD of the tracks in a field of view of a dataset. For the data shown in **(b)** to **(c)**, each dot reports aggregated results from ~>10^3 tracks/field of view/per dataset, collected over 30 or more experiments and 3 or more biological replicates. **(e)** Statistical analysis of results in **(a)** to **(d)**. A Kolmogorov-Smirnov analysis was used to determine whether *D*, %Colocalized and τ_{on} are significantly different ($p<0.01$) from control measurements of EGFR treated with 4 nM EGF at 37°C or not ($p>0.05$). The MSD values at t = 15 s were considered significantly different if the error bars did not overlap.

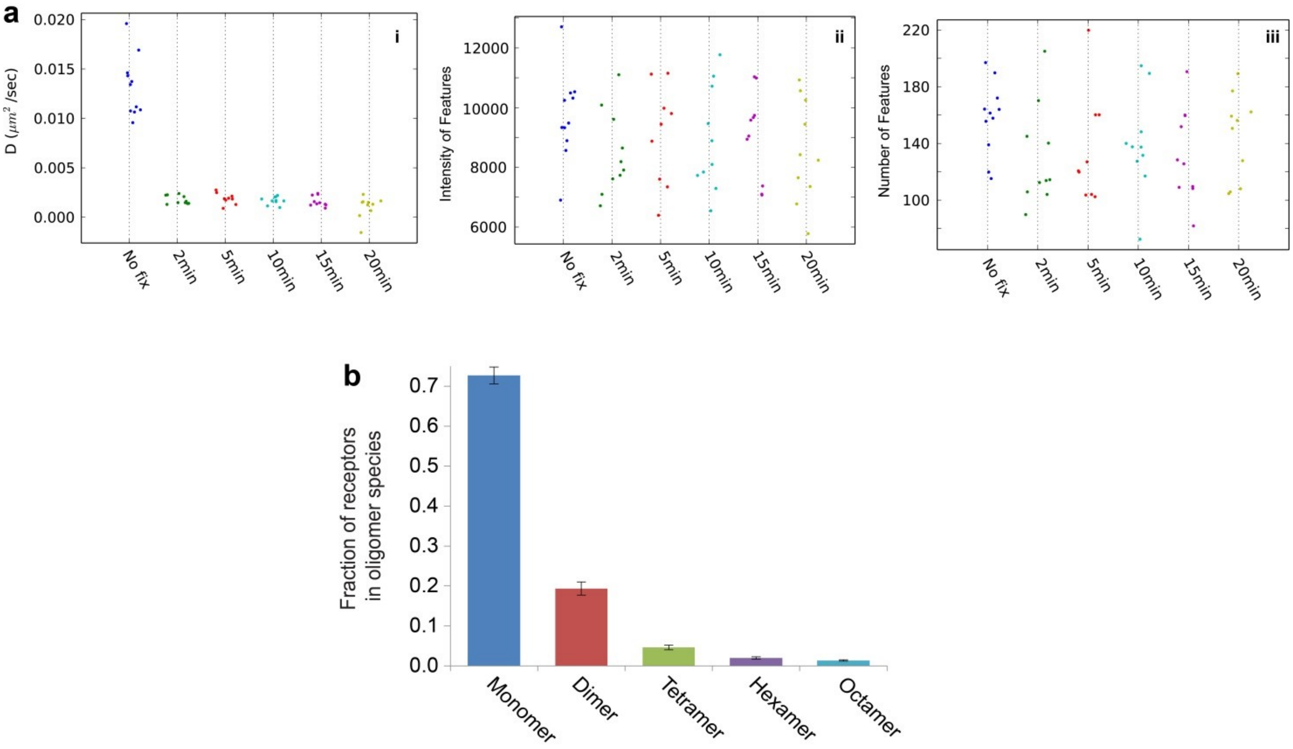

Supplementary Figure 11: Exploring artefacts from chemical fixation. **(a)** i. Instantaneous EGFR particle diffusion rate (*D*) as a function of the time elapsed since a chemical fixative was added at t = 0. *D*. In each figure panel, one dot reports aggregated results from ~>10^3 tracks per field of view per dataset (from 10 individual measurements). Cells were pre-treated with fluorophore-conjugated EGF or 30 minutes at 4°C and kept at 4°C during the different times they were exposed to the fixative, after which they were rinsed thoroughly with PBS. Each point shows the estimate of *D* from a mean-square displacement (MSD) curve of the tracks within a field of view in each dataset. (*Note that complete receptor immobilization was achieved in t < 2 min.*) ii. The average intensity of single particle features detected in each field of view[7] as a function of time since adding the fixative. iii. The number of features detected in each field of view. (*Note the intensity and number of features detected are indistinguishable before and after the fixative*.). **(b)** Concentration-dependent distribution of oligomers species on CHO cells stably expressing EGFR-eGFP mock-treated with PBS, cooled to 4°C and fixed, determined by Photobleaching Image Correlation Spectroscopy (pbICS)[9,10]. Fluorescent images of the labelled receptor at the baso-lateral membrane were repetitively scanned with an Olympus FV1000 laser scanning confocal microscope. The resulting images were subjected to pbICS as described in[9]. For the analysis, a monomer, dimer, tetramer aggregation model was assumed where the fraction of each oligomeric species was variable and allowed to float in the fitting of the pbICS curves. The reported average (and standard deviation) fractions of monomer, dimer and tetramer were obtained from more than ten curves per treatment condition. The results are the mean from three independent biological repeats.

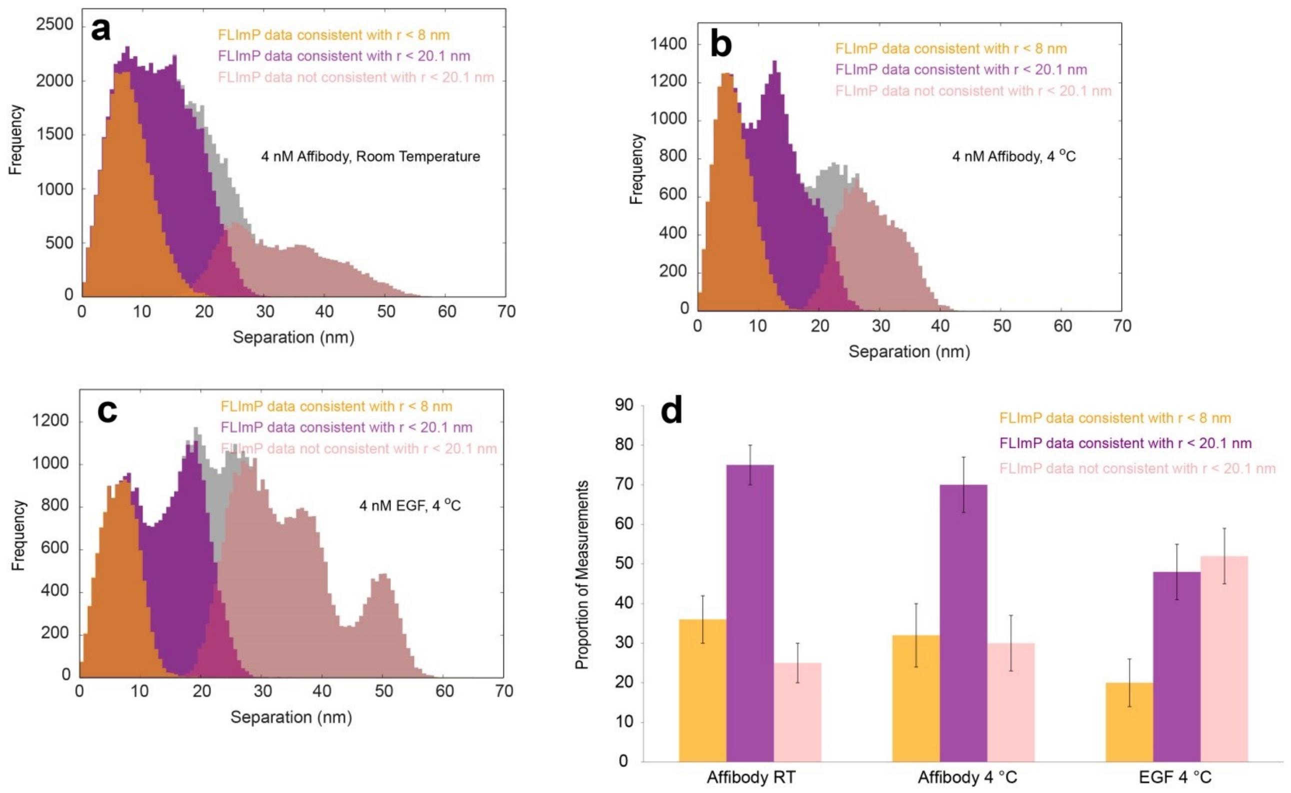

Supplementary Figure 12: Comparison between FLImP distributions at room temperature and low temperature. FLImP distributions (grey) of pairwise separations of fluorophore-conjugated ligand bound to EGFR on CHO cells treated with **(a)** 4 nM Affibody at room temperature (70 FLImP measurements, confidence intervals < 8.0 nm), **(b)** 4 nM Affibody at 4°C (37 measurements, CI < 5.5 nm) , and **(c)** 4 nM EGF at 4°C (51 measurements, CI < 6.0 nm), showing the FLImP measurements whose 69% confidence interval overlaps with the separation range of 0–8 nm (orange), 0-20.1 nm (purple), and not overlapping with either of these ranges (pink). **(d)** Histogram of data from **(a)**, **(b)**, and **(c)** showing proportions of FLImP measurements consistent with the separation ranges described. The FLImP distribution in **(a)** was compiled from 70 FLImP measurements with confidence intervals < 8 nm. Analysis of errors from FLImP measurements is described in **Supplementary Methods.**

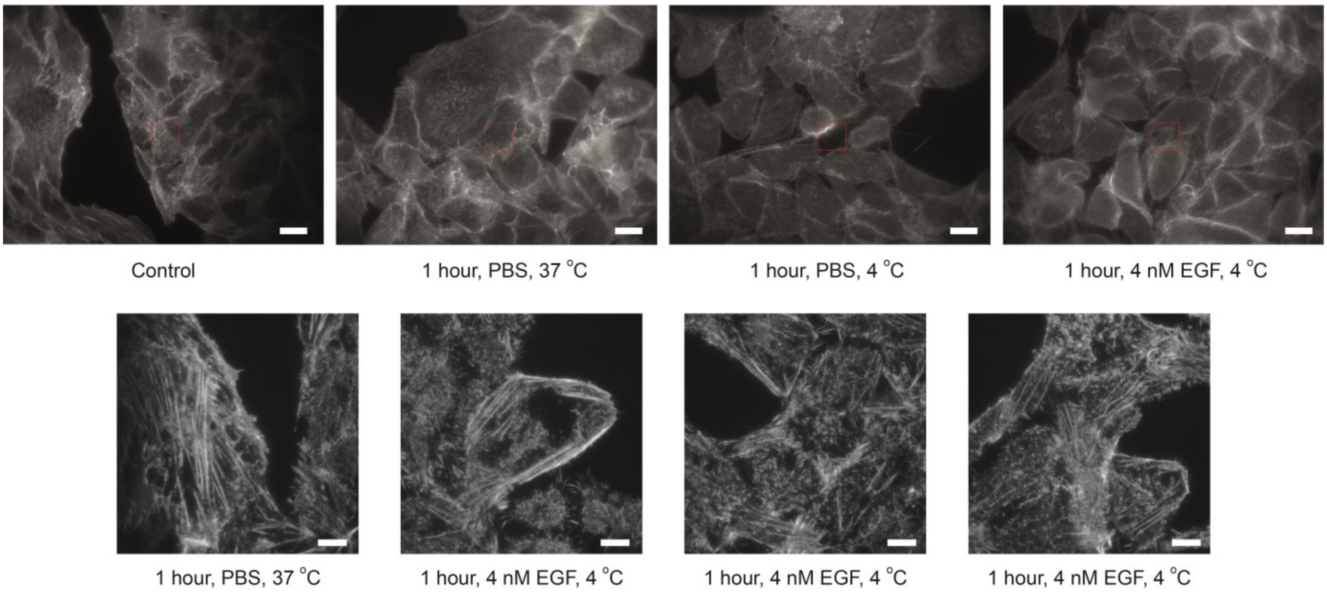

Supplementary Figure 13: Fluorescence microscopy images showing that actin filaments are not disrupted by low temperature treatment. Actin filaments imaged in CHO cells under a range of conditions demonstrating that actin polymerization does not appear to be affected by low temperature treatment. For these experiments, 1 x 10^5 CHO cells were seeded on 1% BSA-coated 35 mm no. 1.5 (high tolerance) glass-bottomed dishes (MatTek Corporation, USA) in 2 ml of media plus 50 ng/ml of doxycycline hyclate (Sigma), resulting in expression of ~10^5 receptors/cell[11]. After two days the medium was changed to 0.1% serum plus 50 ng/ml doxycycline for 2 h. Samples were retrieved from the incubator at 37°C, rinsed with PBS at 37°C, and either fixed immediately for 15 min at room with 3% paraformaldehyde plus 0.5% glutaraldehyde temperature, or incubated in PBS for 1h at 37°C before fixing as just described, or cooled for 10 min on ice at 4°C. The cooled samples were then either further incubated with ice cold PBS or ice cold 4 nM EGF for 1 hour at 4°C in the fridge. The samples were rinsed with PBS and fixed with 3% paraformaldehyde plus 0.5% glutaraldehyde for 15 min at 4°C, then 15 min at room temperature. All samples were rinsed thoroughly with PBS prior to permeabilizing with 0.1% Triton X-100 in PBS for 5 min. Samples were rinsed and labelled with 1U of phalloidin-Alexa647 for 30 min at room temperature and rinsed with PBS. Samples were imaged using either widefield fluorescence microscopy (Vutara SR-350) or TIRF microscopy (Zeiss Axiovert 200M microscope with TIRF illuminator). Scale bars 10 µm.

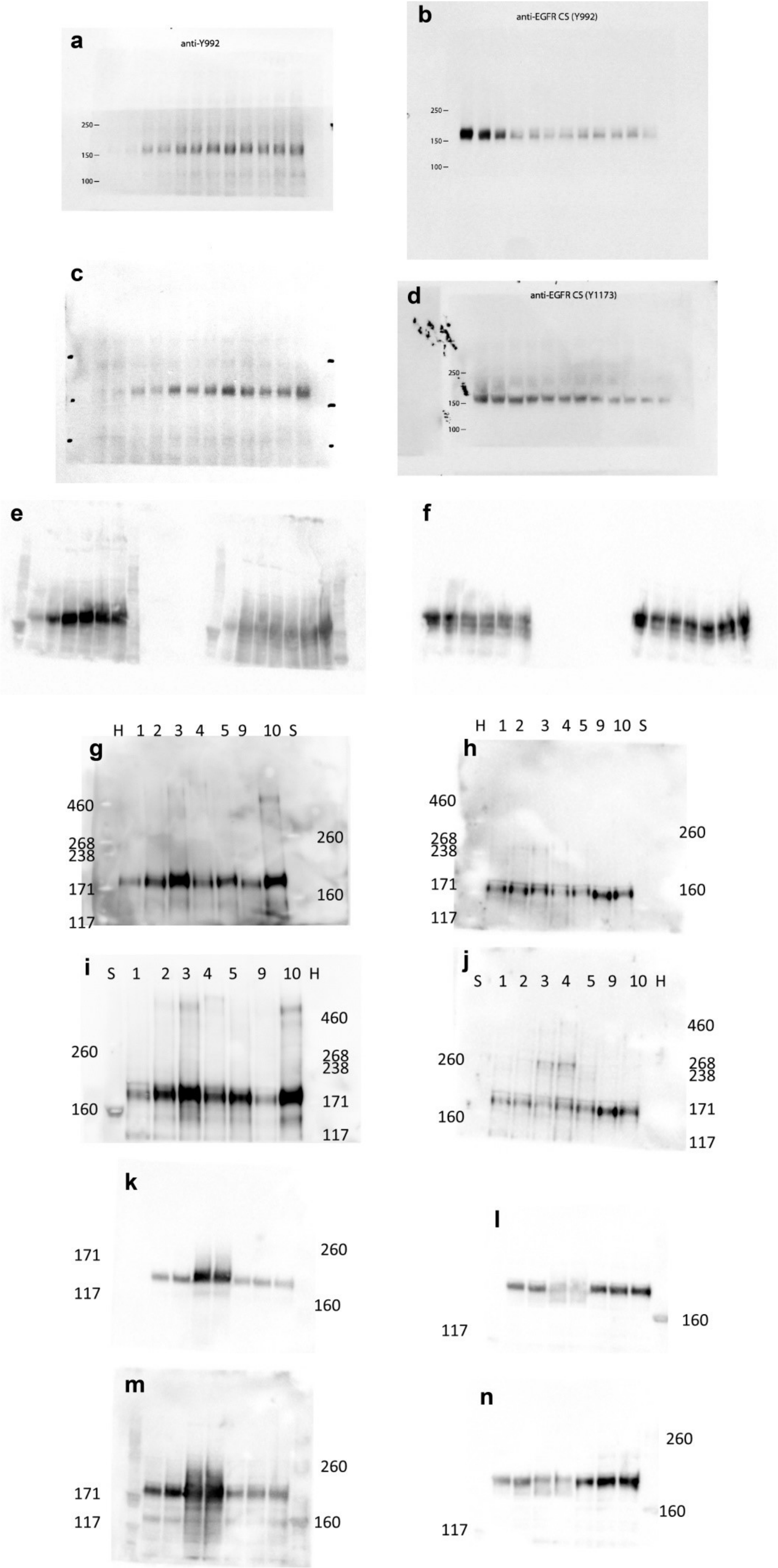
a
anti-Y992
250
150
100
b
anti-EGFR CS (Y992)
250
150
100
c
d
anti-EGFR CS (Y1173)
250
150
100
e
f
g
H 1 2 3 4 5 9 10 S
460
268
238
171
117
260
160
h
H 1 2 3 4 5 9 10 S
460
268
238
171
117
260
160
i
S 1 2 3 4 5 9 10 H
260
160
460
268
238
171
117
j
S 1 2 3 4 5 9 10 H
260
160
460
268
238
171
117
k
171
117
260
160
l
117
160
m
171
117
260
160
n
260
117
160

Supplementary Figure 14: Uncropped Western Blots. (a) and **(b)** uncropped blots shown in **Supplementary Fig. 6a**. Example of phosphorylation of Tyr992 of EGFR detected with mouse anti-EGFR pY992 antibody EM-12, and total EGFR probed with an anti-EGFR cocktail (D38B1, ab137660 and 10005: sc-03). **(c)** and **(d)** blots shown in **Supplementary Fig. 6b**. Example of phosphorylation of Tyr1173 detected with rabbit anti-EGFR pY1173 as a function of EGF dose response, and total EGFR probed with an anti-EGFR cocktail (D38B1, ab137660 and 10005: sc-03). **(e)** and **(f)** blots shown in **Supplementary Fig. 6c**. EGFR phosphorylation on CHO cells expressing wild type EGFR detected with pan-phosphorylation 4G10 antibody as a function of EGF dose response. Total EGFR probed as in **(b)** and **(d). (g)** to **(j)** blots used in **Supplementary Fig. 6d**. A comparison of the total phosphorylation and Tyr1173 phosphorylation between wild type EGFR and the R647/V650C-EGFR mutant on cells treated with 100 nM EGF. **(g)** is top row of crop shown in **Supplementary Fig, 6d** (lanes 1 and 2 and 9 and 10), **(h)** is second row of crop shown in **Supplementary Fig, 6d** (lanes 1 and 2 and 9 and 10), **(i)** is third row of crop shown in **Supplementary Fig, 6d** (lanes 1 and 2 and 9 and 10), and **(j)** is bottom row of crop shown in **Supplementary Fig, 6d** (lanes 1 and 2 and 9 and 10). **(k)** and **(l)** blots used in **Supplementary Fig. 9a**, using pTyr1173-specific antibody **(k)** and an antibody against the EGFR N-terminal tail from CHO cells **(l)** (expressing wild-type EGFR) treated with 1, 10, or 100 nM of Affibody or EGF for 1 hour at 4°C. **(m)** and **(n)** blots used in **Supplementary Fig. 9b**, using the pan-phosphotyrosine antibody 4G10 **(m)** and an antibody against the EGFR N-terminal tail from CHO cells **(n)** (expressing wild-type EGFR) treated with 1, 10, or 100 nM of Affibody or EGF for 1 hour at 4°C.

SUPPLEMENTARY METHODS

Decomposing the FLImP distribution

We make the assumption that the ground truth is an oligomer/fixed assembly of molecules in which there is finite number of discrete distances between the possible labels. Each FLImP separation measurement from the lowest two intensity levels of a spot is therefore a measurement of one of these discrete label–label distances. Each FLImP measurement's bootstrap distribution is therefore a posterior for the underlying separation to which it corresponds, and if we knew a-priori to which each of our discrete separations a measurement corresponds, we could multiply all the posteriors for each separation to get a posterior for that underlying separation. However, for data where the different measurements are not sufficiently separated, we cannot know in advance this assignment of measurements to true separations.
One robust, fully-Bayesian approach is to construct a posterior for the number of discrete distances, the values for these distances, and all possible assignments of measurements to these distances, given the full set of FLImP bootstrap distributions. The optimal number of separations, and their values can then objectively and robustly be estimated. We have attempted this approach, and while it works, it is massively computationally demanding due to the large parameter space, and for the number of measurements in each condition in this paper was beyond the capability of current optimisation methods. We will revisit this in future work.
For this paper we therefore adopted the approach of modelling the FLImP distribution – the sum, $H(r)$, of the individual FLImP measurement bootstrap distributions – as a sum of Rice distributions. $H(r)$ is not a posterior for the separation, it tells us the evidence for a separation r given any of the measurements: it is a histogram of measured separations which accounts for the uncertainty in each.

Justification for Rician model

The FLImP measurement, $\hat{R}_i$, of a particular discrete underlying separation, R_{true}, should be distributed as a Rice distribution,[12] $\mathrm{Pr}\left(\hat{\mathrm{R}}_\mathrm{i}|\mathrm{R}_\mathrm{true}\right) = \mathrm{Rice}\left(\hat{\mathrm{R}}_\mathrm{i}|\mathrm{R}_\mathrm{true}, \sigma\right)$, whose width σ depends on the localisation error of each fluorophore. This is because the localisation error of each fluorophore is well-approximated by a 2D axisymmetric Gaussian distribution, such that the probability of a particular difference in position, $(\hat{x}_i,\, \hat{y}_i)$, being measured is

$$\mathrm{Pr}(\hat{\mathrm{x}}_\mathrm{i}, \hat{\mathrm{y}}_\mathrm{i}|\mathrm{x}_\mathrm{true}, \mathrm{y}_\mathrm{true}) = \mathrm{Gaussian}(\hat{\mathrm{x}}_\mathrm{i}, \hat{\mathrm{y}}_\mathrm{i}|\mathrm{x}_\mathrm{true}, \mathrm{y}_\mathrm{true}, \sigma) = \frac{1}{2\pi\sigma^2}\exp - \frac{(\hat{\mathrm{x}}_\mathrm{i} - \mathrm{x}_\mathrm{true})^2 + (\hat{\mathrm{y}}_\mathrm{i} - \mathrm{y}_\mathrm{true})^2}{2\sigma^2}$$

with $\hat{R}_i = \sqrt{\hat{x_i}^2 + \hat{y_i}^2}$ and $R_{true} = \sqrt{x_{true}^2 + y_{true}^2}$. The distribution of $\hat{R}_i$ for this case is a Rice distribution.
A correctly estimated posterior, $\mathrm{Pr}(\mathrm{x}, \mathrm{y}|\hat{\mathrm{x}}_\mathrm{i}, \hat{\mathrm{y}}_\mathrm{i})$, for each measured position different should also be such as 2D Gaussian,

$$\mathrm{Pr}(\mathrm{x}, \mathrm{y}|\hat{\mathrm{x}}_\mathrm{i}, \hat{\mathrm{y}}_\mathrm{i}) = \mathrm{Gaussian}(\mathrm{x}, \mathrm{y}|\hat{\mathrm{x}}_\mathrm{i}, \hat{\mathrm{y}}_\mathrm{i}, \sigma) = \frac{1}{2\pi\sigma^2}\exp - \frac{(\mathrm{x} - \hat{\mathrm{x}}_\mathrm{i})^2 + (\mathrm{y} - \hat{\mathrm{y}}_\mathrm{i})^2}{2\sigma^2}$$

and so the posterior for the separation, r $(= \sqrt{x^2 + y^2})$, should be a Rice distribution, $\mathrm{Pr}\left(\mathrm{r}|\hat{\mathrm{R}}_\mathrm{i}\right) = \mathrm{Rice}\left(\mathrm{r}|\hat{\mathrm{R}}_\mathrm{i}, \sigma\right)$. Each FLImP bootstrap separation distribution sample is an empirical estimate of this distribution. The sum of N_{meas} FLImP separation (x, y) distributions is then (assuming all measurements have the same error and (x_{true}, y_{true}))

$$H(x, y) = \sum_{i=1}^{N_{meas}} \mathrm{Pr}(\hat{\mathrm{x}}_\mathrm{i}, \hat{\mathrm{y}}_\mathrm{i}|\mathrm{x}_\mathrm{true}, \mathrm{y}_\mathrm{true})\mathrm{Pr}(\mathrm{x}, \mathrm{y}|\hat{\mathrm{x}}_\mathrm{i}, \hat{\mathrm{y}}_\mathrm{i}) \tag{1}$$

$$= \sum_{i=1}^{N_{meas}} \frac{1}{2\pi\sigma^2}\exp - \frac{(\hat{x_i} - x_{true})^2 + (\hat{y_i} - y_{true})^2}{2\sigma^2} \frac{1}{2\pi\sigma^2}\exp - \frac{(x - \hat{x_i})^2 + (y - \hat{y_i})^2}{2\sigma^2} \tag{2}$$

In the limit $N_{meas} \to \infty$ this becomes a convolution of the two Gaussians, which is a Gaussian of variance $2\sigma^2$ centred on (x_{true}, y_{true}).

$$\lim_{N_{meas}\to\infty} H(x, y) = \frac{1}{2\pi(\sqrt{2}\sigma)^2}\exp - \frac{(x - x_{true})^2 + (y - y_{true})^2}{2(\sqrt{2}\sigma)^2} \tag{3}$$

and so for r we will have a Rice distribution for variance $2\sigma^2$

$$\lim_{N_{meas}\to\infty} H(r) = \mathrm{Rice}\left(\mathrm{r}|\mathrm{R}_\mathrm{true}, \sqrt{2}\sigma\right) \tag{4}$$

In reality each different FLImP measurement will have a different orientation in x-y, but rotating the x-y axes for each measurement so that Equation 3 holds does not affect the distribution of r. The finite number of measurements and the

range of localisation errors and therefore different values of σ means the true empirical $H(r)$ is not a perfect Rician, as illustrated in the simulations in **Supplementary Fig. 3a**, but it is still a suitable approximation with our choice of confidence intervals discussed later.
In the case that there are N_{comp} true underlying separations, $R_{true,j}$, present with proportions w_j, with $\sum_{j=1}^{N_{comp}} w_j = 1$ we have the model

$$H(r|\boldsymbol{w}, \boldsymbol{R_{true}}, \boldsymbol{\sigma}, N_{comp}) = \sum_{j=1}^{N_{comp}} w_j \mathrm{Rice(r|R_{true,j}, \sigma_j)} \tag{5}$$

where we allow each component to have a different standard deviation, σ_j, to allow for the possibility of measurements with different localisation errors contributing to each. $\boldsymbol{w} = [w_1, w_2, .., w_{N_{comp}}]$, $\boldsymbol{R_{true}} = [R_{true,1}, R_{true,2}, .., R_{true,N_{comp}}]$ and $\boldsymbol{\sigma} = [\sigma_1, \sigma_2, .., \sigma_{N_{comp}}]$.

Parameter estimation and model selection

Having adopted the sum of Rician components in equation 5 as a model for the sum of FLImP distributions, we must now fit this model to our measured set of FLImP distributions. Each FLImP measurement (index i_{meas}) separation distribution is a sample of N_{Fboot} separation values, $\boldsymbol{r}_{i_{meas}} = [r_{i_{meas},1}, r_{i_{meas},2}..r_{i_{meas},N_{Fboot}}]$, which is considered to be a sample representative of the posterior of the separation for that measurement. If we pool the N_{meas} $\boldsymbol{r}$ measurement samples for a particular condition (with each sample having the same N_{Fboot} to give each measurement equal weighting), then $H(r)$ is a likelihood for each of those pooled sample values, so that the likelihood for measuring the FLImP samples given the model is

$$L(\boldsymbol{r}_1, \boldsymbol{r}_2, .., \boldsymbol{r}_{N_{meas}}|\boldsymbol{w}, \boldsymbol{R_{true}}, \boldsymbol{\sigma}, N_{comp}) = \prod_{i_{meas}=1}^{N_{meas}} \prod_{j=1}^{N_{Fboot}} H(r_{i_{meas},j})$$

For our data $N_{Fboot} = 1200$, and N_{meas} is in the range 30–50. To speed up the calculation of L we therefore binned the pooled $\boldsymbol{r}$ into a uniform grid of N_{bins} separation bins, where bin $ibin$ has separation r_{ibin} and frequency f_{ibin}. We then have

$$L(\boldsymbol{r}_1, \boldsymbol{r}_2, .., \boldsymbol{r}_{N_{meas}}|\boldsymbol{w}, \boldsymbol{R_{true}}, \boldsymbol{\sigma}, N_{comp}) = \prod_{ibin=1}^{N_{bins}} H(r_{ibin})^{f_{ibin}}$$

typically reducing the number of $H(r)$ evaluations by a factor ~ 500. By setting the grid spacing to 2.5 bins per standard deviation of the narrowest FLImP measurement distribution, this binning resolves well the distribution of measurements and so does not affect the result.
We applied a prior $\Pi(\boldsymbol{w}, \boldsymbol{R_{true}}, \boldsymbol{\sigma}|N_{comp}) = \Pi_{\boldsymbol{w}}\Pi_{\boldsymbol{R_{true}}}\Pi_{\boldsymbol{\sigma}}$ to impose constraints on the parameters as follows:

- We set $\Pi_{\boldsymbol{w}} = 1$ if $\frac{w_j}{\sum_{j=1}^{N_{comp}} w_j} > 0.9/N_{meas}$ for all j, and $\Pi_{\boldsymbol{w}} = 0$ otherwise. This ensures that no component in the model can correspond to fewer than 0.9 measurements. In principle none should be less than one measurement so this allows for a small margin of error.
- We set $\Pi_{\boldsymbol{w}} = 1$ if $min(\boldsymbol{\sigma_{FLImP}}) \leq \sigma_j \leq \sqrt{2} \times max(\boldsymbol{\sigma_{FLImP}})$ for all j, and $\Pi_{\boldsymbol{\sigma}} = 0$ otherwise, where $\boldsymbol{\sigma_{FLImP}}$ is the list of maximum likelihood Rician fit σs of each included FLImP separation distribution. The lower limit arises because the narrowest peak possible is a component corresponding to just the FLImP measurement with the smallest confidence interval. The upper limit arises because the widest possible should contain multiple measurements at the upper end of the measurement confidence interval, and so from equation 4 have a σ which is $\sqrt{2}$ times $max(\boldsymbol{\sigma_{FLImP}})$.
- We also forced each $R_{true,j}$ to be in the range R_{min} to R_{max} and in ascending order ($R_{true,j} > R_{true,j-1}$). This was done by reparameterising R_{true} as $R'_{true,j} = \frac{R_{true,j} - R_{true,j-1}}{R_{max} - R_{true,j-1}}$ with $R_{true,0} = R_{min}$, setting $\Pi_{\boldsymbol{R_{true}}} = 1$ if $0 \leq R'_{true,j} \leq 1$ for all j and $\Pi_{\boldsymbol{R_{true}}} = 0$ otherwise. We used R_{min}=0 nm and R_{max}=60 nm.

We then optimised the unnormalised posterior,

$$\mathrm{Pr}\big(\boldsymbol{w}, \boldsymbol{R'_{true}}, \boldsymbol{\sigma}|\boldsymbol{r}_1, \boldsymbol{r}_2, .., \boldsymbol{r}_{\mathrm{N_{meas}}}, \mathrm{N_{comp}}\big) = \mathrm{L}(\boldsymbol{r}_1, \boldsymbol{r}_2, .., \boldsymbol{r}_{\mathrm{N_{meas}}}|\boldsymbol{w}, \boldsymbol{R'_{true}}, \boldsymbol{\sigma}, \mathrm{N_{comp}})\Pi(\boldsymbol{w}, \boldsymbol{R'_{true}}, \boldsymbol{\sigma}|\mathrm{N_{comp}})$$

using the downhill simplex method[13] to find the most probable values of $\boldsymbol{w}$, $\boldsymbol{R'_{true}}$ (and therefore $\boldsymbol{R_{true}}$) and $\boldsymbol{\sigma}$ given the data, $\hat{\boldsymbol{w}}$, $\hat{\boldsymbol{R}}_{\boldsymbol{true}}$, $\hat{\boldsymbol{\sigma}}$
This is a highly multidimensional and multi-modal problem. To ensure identification of the global optimimum we found that repeating the fit 800 times and choosing the result with the maximum posterior value produced consistent results.
The likelihood used for this calculation does not account for the effect on $H(r)$ of the discrepancy between the perfect sum of Ricians assumed and the reality of a sum of a finite-number of FLImP distributions. Explicity incorporating this into the likelihood would be challenging. Instead we repeat the whole fitting process 50 times for each dataset, each time using a different synthetic dataset produced from the true dataset by resampling with replacement the FLImP measurements to be

included. The distribution of fitted parameters obtained from these bootstrap-resampled datasets then tells about their uncertainty as a result of the finite number of measurements and their distribution.[13]

A key question to address is how many components we should use when performing the model selection. For this we used the Bayesian information criterion, $BIC = -2\ln\hat{L} + k\ln n$, where $\hat{L}$ is $L(\hat{\boldsymbol{w}}, \hat{\boldsymbol{R}}_{\mathbf{true}}, \hat{\boldsymbol{\sigma}})$, the likelihood at the most probably parameters, k is the number of free parameters, $3 * N_{comp} - 1$ (-1 because the constraint $\sum_{j=1}^{N_{comp}} w_j = 1$ removes one free parameter), and n is the number of data points, $N_{meas} \times N_{Fboot}$. A model with lower BIC is preferred, with an improvement in $BIC \sim 10$ considered significant.[14] We therefore performed our fit for a range of N_{comp}, and compared the BIC for each. Since we did not explicitly include the effect of finite number of samples in our likelihood, simply using this BIC will not fully account for the deviation between the model and the true data, potentially leading to overfitting of the data. However, as described above, we fitted each model another 50 times using bootstrap resampled dataset to assess the effect of the finite number of samples on our result. Therefore the consistency of model selection conclusions across the $BIC(N_{comp})$ curves for the resampled datasets accounts for the effect of finite-counting on the model selection. For each N_{comp} we determined the proportion of BIC curves for which that N_{comp} was significantly better than for all lower N_{comp} in the corresponding curve. We then chose the model with the highest N_{comp} for which this proportion was at least 50%, ie. we chose the model with the largest N_{comp} which is more likely than not to be significantly better than all simpler (lower N_{comp}) models. This is illustrated in **Supplementary Fig. 3b**. In a few cases the best model found this way has a the minimum allowed peak weight $0.9/N_{meas}$), indicating that the parameter optimisation really wanted to make it zero. In these cases we then chose the next best model with lower N_{comp}.

The final question is how to interpret the result of this in terms of our discrete separation model. We have not fitted here a posterior of the discrete separations, we have fitted a sum of Ricians as a model for the sum of our histograms. However in section we justified this model $H(r)$ given the hypothesis of discrete separations. To use the fitted Rice distribution parameters $\hat{\boldsymbol{w}}$, $\hat{\boldsymbol{R}}_{\mathbf{true}}$, $\hat{\boldsymbol{\sigma}}$ to estimate the true model discrete separation, we performed simulations of typical data and identified appropriate confidence intervals to use. This is non-trivial for a Rice distribution due to its assymmetry and properties when the separation and localisation error are comparable. In particular, even if the true separation is 0, the likelihood of measuring a separation 0 is 0. This leads to biases and overestimation of short separations. We found (**Supplementary Fig. 1d**) that for simulations representative of our data, $\hat{R}_{true}$ for peaks with separation less than $\sim 5\,\text{nm}$ can be over- or underestimated by several nm. In particular an overestimate in the worst case of 3nm is possible for a true separation of 0nm and a peak with only 1 measurement. We chose to use the most compact confidence interval in r, $(\hat{R}_{true,j,peak,low}, \hat{R}_{true,j,peak,high})$ which includes both $\hat{R}_{true}$ and 69% of a Rice distribution $\mathrm{Rice}\left(\mathrm{r}|\hat{\mathrm{R}}_{\mathrm{true,j}}, \sigma_{\mathrm{CI,j}}\right)$, with $\sigma_{CI,j} = \sigma_j$ for $\hat{R}_{true} \leq 5.5\text{nm}$ and $\sigma_{CI,j} = \hat{\sigma}_j/\sqrt{\hat{w}_j N_{meas}}$ for $\hat{R}_{true} > 5.5\text{nm}$. We explicitly reduced $\hat{R}_{true,j,low}$ by 2.5nm for peaks with $\hat{w}_j N_{meas} \leq 5$ to account for the most extreme overestimate. Our simulations show that these intervals are robust to at least 75% for representative simulations. (**Supplementary Fig. 1e**).

To account for the error in the decomposition of the positions due to the finite number of measurements, we use the distribution, $\hat{R}_{true,j,boot}$ of the $\hat{R}_{true,j}$ from the fits to the bootstrap-resampled data. We work out the width, $CI_{R_{true},boot}$, of the most compact interval,$(\hat{R}_{true,j,boot,low}, \hat{R}_{true,j,boot,high})$, containing 69% of the values in $\hat{R}_{true,j,boot}$. We then extend this using the CI from the best fit peaks to the true dataset to give final confidence intervals, $(\hat{R}_{true,j,low}, \hat{R}_{true,j,high})$, with

$$\hat{R}_{true,j,low} = \hat{R}_{true,j,boot,low} + \hat{R}_{true,j,peak,low} - \hat{R}_{true,j}$$

and

$$\hat{R}_{true,j,high} = \hat{R}_{true,j,boot,high} + \hat{R}_{true,j,peak,high} - \hat{R}_{true,j}$$

Supplementary Fig. 3b shows an example of the process for simulated data.

SUPPLEMENTARY REFERENCES

1 Needham, S. R. *et al.* Determining the geometry of oligomers of the human epidermal growth factor family on cells with < 10 nm resolution. *Biochemical Society Transactions* **43**, 309-314, doi:10.1042/bst20140318 (2015).

2 Ogiso, H. *et al.* Crystal structure of the complex of human epidermal growth factor and receptor extracellular domains. *Cell* **110**, 775-787 (2002).

3 Kalinin, S. *et al.* A toolkit and benchmark study for FRET-restrained high-precision structural modeling. *Nat Methods* **9**, 1218-1225, doi:10.1038/nmeth.2222 (2012).

4 Press, W. H., Teukolsky, S. A., Vetterling, W. T. & Flannery, B. P. (University of Cambridge, New York, USA, 1996).

5 Needham, S. R. *et al.* Measuring EGFR Separations on Cells with similar to 10 nm Resolution via Fluorophore Localization Imaging with Photobleaching. *PLoS One* **8**, doi:10.1371/journal.pone.0062331 (2013).

6 Tynan, C. J. *et al.* Human Epidermal Growth Factor Receptor (EGFR) Aligned on the Plasma Membrane Adopts Key Features of Drosophila EGFR Asymmetry. *Mol Cell Biol* **31**, 2241-2252, doi:10.1128/mcb.01431-10 (2011).

7 Rolfe, D. J. *et al.* Automated multidimensional single molecule fluorescence microscopy feature detection and tracking. *Eur Biophys J* **40**, 1167-1186, doi:10.1007/s00249-011-0747-7 (2011).

8 Webb, S. E., Needham, S. R., Roberts, S. K. & Martin-Fernandez, M. L. Multidimensional single-molecule imaging in live cells using total-internal-reflection fluorescence microscopy. *Opt Lett* **31**, 2157-2159 (2006).

9 Ciccotosto, G. D., Kozer, N., Chow, T. T. Y., Chon, J. W. M. & Clayton, A. H. A. Aggregation Distributions on Cells Determined by Photobleaching Image Correlation Spectroscopy. *Biophys J* **104**, 1056-1064, doi:10.1016/j.bpj.2013.01.009 (2013).

10 Kozer, N. *et al.* Exploring higher-order EGFR oligomerisation and phosphorylation-a combined experimental and theoretical approach. *Molecular Biosystems* **9**, 1849-1863, doi:10.1039/c3mb70073a (2013).

11 Macdonald, J. L. & Pike, L. J. Heterogeneity in EGF-binding affinities arises from negative cooperativity in an aggregating system. *Proc Natl Acad Sci U S A* **105**, 112-117, doi:10.1073/pnas.0707080105 (2008).

12 Churchman, L.S. *et al.* A non-Gaussian distribution quantifes distances measured with fluorescence localization techniques. *Biophys. J.* **90**, 668-671 (2006).

13 Press, W.H. *et al. Numerical Recipes in C. The Art of Scientifc Computing.* Cambridge University Press (1992).

14 Kass, R.E., and Raftery, A.E. Bayes Factors. *J. Am. Stat. Assoc.* **90**, 773-795 (1995).
